# Evolution of Zika virus in *Rag1*-deficient mice selects for unique envelope glycosylation motif mutants that show enhanced replication fitness

**DOI:** 10.1093/ve/veaf021

**Published:** 2025-04-11

**Authors:** Eri Nakayama, Bing Tang, Romal Stewart, Abigail L Cox, Kexin Yan, Cameron R Bishop, Troy Dumenil, Wilson Nguyen, Andrii Slonchak, Julian Sng, Alexander A Khromykh, Viviana P Lutzky, Daniel J Rawle, Andreas Suhrbier

**Affiliations:** Infection and Inflammation Department, QIMR Berghofer Medical Research Institute, Brisbane, QLD 4029, Australia; Department of Virology I, National Institute of Infectious Diseases, Shinjuku City, Tokyo 162-0052 Japan; Infection and Inflammation Department, QIMR Berghofer Medical Research Institute, Brisbane, QLD 4029, Australia; Infection and Inflammation Department, QIMR Berghofer Medical Research Institute, Brisbane, QLD 4029, Australia; Infection and Inflammation Department, QIMR Berghofer Medical Research Institute, Brisbane, QLD 4029, Australia; Infection and Inflammation Department, QIMR Berghofer Medical Research Institute, Brisbane, QLD 4029, Australia; Infection and Inflammation Department, QIMR Berghofer Medical Research Institute, Brisbane, QLD 4029, Australia; Infection and Inflammation Department, QIMR Berghofer Medical Research Institute, Brisbane, QLD 4029, Australia; Infection and Inflammation Department, QIMR Berghofer Medical Research Institute, Brisbane, QLD 4029, Australia; School of Chemistry and Molecular Biosciences, The University of Queensland, St Lucia, QLD 4072, Australia; Australian Infectious Disease Research Centre, GVN Center of Excellence, Brisbane, QLD 4029 and 4072, Australia; School of Chemistry and Molecular Biosciences, The University of Queensland, St Lucia, QLD 4072, Australia; School of Chemistry and Molecular Biosciences, The University of Queensland, St Lucia, QLD 4072, Australia; Australian Infectious Disease Research Centre, GVN Center of Excellence, Brisbane, QLD 4029 and 4072, Australia; Infection and Inflammation Department, QIMR Berghofer Medical Research Institute, Brisbane, QLD 4029, Australia; Infection and Inflammation Department, QIMR Berghofer Medical Research Institute, Brisbane, QLD 4029, Australia; Infection and Inflammation Department, QIMR Berghofer Medical Research Institute, Brisbane, QLD 4029, Australia; Australian Infectious Disease Research Centre, GVN Center of Excellence, Brisbane, QLD 4029 and 4072, Australia

**Keywords:** Zika virus, glycosylation, *Rag1* ^-/-^ mice, brain organoid, neutralizing antibody

## Abstract

N-linked glycosylation of flavivirus envelope proteins is widely viewed as being required for optimal folding, processing and/or transit of envelope proteins, and the assembling virons, through the endoplasmic reticulum (ER) and the Golgi. Zika virus (ZIKV) has a single N-linked envelope glycan located adjacent to the fusion loop. Herein we show that independent serial passage of ZIKV_Natal_ in *Rag1*^-/-^ mice for 223 or 386 days generated two unique envelope glycan-deficient mutants, ZIKV-V153D and ZIKV-N154D, respectively. Surprisingly, these mutants grew to titres ∼1 to 2.6 logs higher than the glycosylated parental ZIKV_Natal_ in Vero E6 cells and human brain organoids. RNA-Seq of infected organoids suggested that this increased replication fitness was associated with upregulation of the unfolded protein response (UPR). Cell death, cellular viral RNA, and viral protein levels were not significantly affected, arguing that these glycan mutants enjoyed faster ER/Golgi folding, processing, assembly, transit, and virion egress, assisted by an upregulated UPR. Thus, ZIKV envelope N-linked glycosylation is not essential for promoting envelope folding, assembly, and transit through the ER/Golgi, since aspartic acid (D) substitutions in the glycosylation motif can achieve this with significantly greater efficiency. Instead, the evolution of glycan mutants in *Rag1*^-/-^ mice indicates that such envelope glycosylation can have a fitness cost in an environment devoid of virus-specific antibody responses. The V153D and N154D mutations, generated by natural selection in *Rag1*^-/-^ mice, have to date not been employed in orthoflavivirus envelope glycosylation studies. Instead, genetic engineering has been used to generate mutant viruses that, for instance, contain a N154A substitution. The latter may impart confounding unfavourable properties, such as envelope protein insolubility, that have a detrimental impact on virus replication. The V153D and N154D substitutions may avoid imparting unfavourable properties by preserving the surface negative charge provided by the glycan moiety in the parental ZIKV_Natal_ envelope protein. In *Ifnar1*^-/-^ mice ZIKV-V153D and -N154D showed faster viremia onsets, but reduced viremic periods, than the parental ZIKV_Natal_, consistent with an established contention that such glycans have evolved to delay neutralizing antibody activity.

## Introduction

Zika virus (ZIKV) is a positive sense, single-stranded RNA arbovirus that belongs to the *Flavivirus* genus (renamed *Orthoflavivirus* in 2023) in the family *Flaviviridae*, and is primarily transmitted to humans by *Aedes aegypti* and *Aedes albopictus* mosquitoes. ZIKV emerged as an important pathogen in 2007, with outbreaks in the Pacific Islands and subsequent spread across the Americas in 2015–16 (Saba Villarroel et al. 2024). The World Health Organization (WHO) regards ZIKV as a priority disease for research and development after declaring a public health emergency of international concern in 2016. ZIKV infection of pregnant women can lead to congenital Zika syndrome (CZS), which comprises a spectrum of primarily neurological disorders in neonates (Freitas et al. 2020; Moura et al. 2021). CZS can be associated with severe functional impairments that have profound adverse impacts on child development and quality of life (Marques et al. 2024). CZS is also associated with an increased risk of childhood mortality (Paixao et al. 2022). After ZIKV is introduced by the bite of an infected mosquito, viral dissemination and viremia in humans is primarily driven by infection of subsets of monocytes and macrophages (Reynoso et al. 2023). ZIKV can then cross the placenta and enter the developing foetal brain, perhaps via transcytosis across the endothelial cells comprising the blood–brain barrier (Nakayama et al. 2021a; Henrio Marcellin and Huang 2024). Once in the brain, ZIKV can infect a number of brain cell types, including neural progenitor cells, a critical cell population in the developing embryonic brain. Infection of these cells results in growth perturbations, inhibition of differentiation, and apoptosis, all key processes in driving the brain damage associated with CZS (Nakayama et al. 2021b; Komarasamy et al. 2022).

The envelope glycoprotein of ZIKV, and other orthoflaviviruses, is responsible for viral entry into host cells and is a key determinant of viral pathogenesis (Fontes-Garfias et al. 2017; Goo et al. 2017; Giraldo et al. 2020; Cheng et al. 2022), as well as being the target of protective neutralizing antibodies (Rawle et al. 2024). The envelope proteins of nearly all orthoflaviviruses have N-linked glycosylation sites, which for ZIKV is a single glycosylation site located at amino acid N^154^. The ZIKV envelope glycan moiety is adjacent to the fusion loop in the mature virion, and has been proposed to reduce access of neutralizing antibodies targeting the fusion loop region (Sirohi et al. 2016; Frumence et al. 2019; Contreras et al. 2024). However, glycan moieties on viral envelope proteins are reported to have a range of activities (Courageot et al. 2000; Feng et al. 2022), with a large body of literature arguing that loss of N-linked glycans on orthoflaviviral envelope proteins reduces *in vitro* replication, often leading to reduced virulence in animal models (Supplementary Table 1). Other studies show no change or mixed effects (Supplementary Table 1). The endoplasmic reticulum (ER) is arguably the critical hub for orthoflavivirus replication (Verhaegen and Vermeire 2024). A key mechanism proposed to underpin the aforementioned observations is that N-linked glycosylation of the orthoflavivirus envelope protein supports proper folding, processing, stability, maturation and/or transport of envelope, and the assembling virion, during the journey through the ER and Golgi, and the eventual egress into the extracellular milieu (Courageot et al. 2000; Mossenta et al. 2017; Gwon et al. 2020; Ishida et al. 2023; Bishop et al. 2024; Pandey et al. 2025). In contrast, a number of publications report increased replication and/or virulence associated with the loss of N-linked glycosylation of orthoflaviviral envelope proteins (Lee et al. 1997; Chambers et al. 1998; Kariwa et al. 2013; Guo et al. 2021; Fang et al. 2022) or find no, or mixed, effects (Moudy et al. 2009, 2011; Alen et al. 2012; Frumence et al. 2019; Maharaj et al. 2019; Cheng et al. 2022; Zhang et al. 2023b) (Supplementary Table 1). The disparity between these studies may arise, at least in part, from the variety of strategies used to disrupt the N-glycosylation motif (Supplementary Table 1), which for ZIKV usually has the amino acid sequence ^153^VNTD^156^ (Annamalai et al. 2017), although the inclusion of V^153^ in this motif has only been proposed (Gong et al. 2018; Frumence et al. 2019; Gallichotte et al. 2023). A number of orthoflavivirus studies have engineered a N154A substitution to prevent N-link glycosylation at this site (Annamalai et al. 2017; Liang et al. 2018; Liu et al. 2021; Ishida et al. 2023), whereas others have engineered different substitutions or deletions in the glycosylation motif (Prow et al. 2011; Widman et al. 2017; Guo et al. 2021; Cheng et al. 2022) (Supplementary Table 1). A problem of interpretation emerges as either the loss of the glycan moiety and/or the change in the envelope amino acid sequence may be responsible for the effects on virus replication. For instance, the N154A substitution has been associated with reduced solubility of ZIKV envelope protein, with an ensuing rapid degradation of the mutant envelope protein by the host ER-associated degradation system (Ishida et al. 2023). The N154A substitution also increased the toxicity of ZIKV envelope protein expressed in neuronal cells *in vitro* (Steiner et al. 2023). Thus for the N154A mutant virus, it is unclear whether the loss of the glycan is responsible for impaired replication, and/or whether the introduction of the small hydrophobic amino acid, alanine, imparts confounding unfavourable properties on the envelope protein.

ZIKV replication in wild-type mice is inefficient, with mouse model work thus often involving use of interferon (IFN)α/β receptor deficient (*Ifnar1^-/-^*) mice (Lazear et al. 2016; Prow et al. 2018; Setoh et al. 2019; Nakayama et al. 2021b). We previously reported infection of *Ifnar1^-/-^* mice with an African lineage virus isolate, maintained at the National Institute of Infectious Diseases (Tokyo, Japan), ZIKV-MR766-NIID (Nakayama et al. 2021a). This virus isolate does not have an N-linked glycosylation in the envelope protein due to a D156I substitution in the glycosylation motif, VNDI. However, within 4 days post infection (dpi), virus recovered from the serum of *Ifnar1^-/-^* mice showed restoration of the glycosylation motif (VNDT) (Nakayama et al. 2021a). *Ifnar1^-/-^* mice are fully capable of generating neutralizing antibody responses against ZIKV (Prow et al. 2018; Hazlewood et al. 2020). IgM can also neutralize ZIKV (Calvert et al. 2021; Singh et al. 2022) and antigen-specific IgM can appear as early as 1–3 dpi (Lawson et al. 1988; Zhang and Summers 2004; Al-Ibraheemi and Al-Saeedi 2021). This selection for the N-linked envelope glycan may thus have arisen to avoid neutralizing antibody responses (Sirohi et al. 2016; Frumence et al. 2019; Contreras et al. 2024). However, selection may also have occurred because the glycan is required for optimal folding, processing and/or traffic of envelope through the ER. Also possible is that the glycan moiety is selected because it promotes infection of host cells via lectin attachment factors (Carbaugh et al. 2020). RNA viruses (including ZIKV) generally have RNA-dependent RNA polymerases that lack proof-reading ability, leading to high mutation rates and substantial ensuing genetic variation within a replicating virus population (Van Boheemen et al. 2017). Viral mutants that emerge after selection may thus already exist as minor populations in the initial viral inocula and/or may arise *de novo* during the selection process.

To generate more insights into the role of the ZIKV N-linked envelope glycan, and perhaps inform the aforementioned issues, we sought to determine what changes might evolve in mice in the absence of antibody responses. *Rag1*^-/-^ mice were chosen as they are unable to mount adaptive immune responses, but can nevertheless support ZIKV replication (Gorman et al. 2018; Hayashida et al. 2019), despite intact type I IFN responses. Murine type I IFN responses effectively blunt ZIKV replication (Kumar et al. 2016; Grant et al. 2016a); however, antibodies are needed to clear the viremia (Magnani et al. 2017). We chose to use ZIKV_Natal_ (Setoh et al. 2017) as this Asian lineage isolate is unequivocally associated with a human case of microcephaly and has only a brief passage history in C6/36 cells (Mlakar et al. 2016). In *Ifnar1^-/-^* mice, viral titres reached by ZIKV_Natal_ infections are generally lower than for ZIKV-MR766 (Prow et al. 2018; Nakayama et al. 2021a), with infections generally asymptomatic and nonlethal (Setoh et al. 2017). In contrast, ZIKV-MR766 viruses have been extensively passaged in young mice and are usually more virulent (Shao et al. 2017; Nakayama et al. 2021a). Herein, ZIKV_Natal_ was passaged five times in *Rag1*^-/-^ mice in three independent passage series. Remarkably, each passage series selected a virus with mutations in the envelope protein glycosylation motif. Two of these, ZIKV-V153D and ZIKV-N154D, generated titres ∼1 to 2.6 logs higher than the glycosylated parental ZIKV in Vero E6 cells and in human cortical brain organoids. These results argue against a critical role for glycans for folding, processing, assembly, and/or transit of envelope through the ER, as this was clearly achieved with much greater efficiency by the aforementioned aspartic acid substitutions. In *Ifnar1^-/-^* mice, viremia peaked earlier, but the viremic period was shorter, for the two glycan mutants, when compared with the parental ZIKV_Natal_. These observations support the previously expressed contention that such glycans have evolved to delay/avoid generation of, and targeting by, neutralizing antibody responses (Sanders et al. 2008; Martina et al. 2023; Contreras et al. 2024; Zhang et al. 2024; Chen et al. 2025).

## Material and Methods

### Ethics statement and regulatory compliance

All mouse work was conducted in accordance with the ‘Australian code for the care and use of animals for scientific purposes’ as defined by the National Health and Medical Research Council of Australia. Mouse work was approved by the QIMR Berghofer Medical Research Institute Animal Ethics Committee (P2195, A1604-611M and P3746, A2108-612). Mice were euthanized using CO_2_. Breeding and use of GM mice was approved under a Notifiable Low Risk Dealing (NLRD) Identifier: NLRD_Suhrbier_Oct2020: NLRD 1.1(a). All work was approved by the QIMR Berghofer Medical Research Institute Safety Committee (P2195 and P3746). Details of mouse agistment conditions have been described in detail previously (Yan et al. 2022).

### Cell lines and viral titrations

Vero cells (ATCC#: CCL-81) and C6/36 cells (ATCC# CRL-1660) were maintained in RPMI 1640 (Thermo Fisher Scientific, Scoresby, VIC, Australia) supplemented with endotoxin free 10% heat-inactivated foetal bovine serum (Sigma-Aldrich, Castle Hill, NSW, Australia) at 37°C and 5% CO_2_. Cells were checked for mycoplasma using MycoAlert Mycoplasma Detection Kit (Lonza, Basel, Switzerland).

ZIKV titres were determined by focus-forming unit (ffu) assays (Setoh et al. 2019), or by CCID_50_ assays using ten-fold serial dilution titrations, in duplicate in 96-well plates as described (Nakayama et al. 2021a). For the latter, virus titres were determined by the method of Spearman and Karber (https://www.klinikum.uni-heidelberg.de/zentrum-fuer-infektiologie/molecular-virology/welcome/downloads).

### Generation of ZIKV_Natal_

The complete genome sequence for ZIKV_Natal_ was obtained from foetal brain of a human case of microcephaly (Mlakar et al. 2016) (GenBank accession number KU527068). Infectious virus was reconstructed using reverse genetics to produce a primary ZIKV_Natal_ isolate, with no mouse passage history (Setoh et al. 2017). ZIKV_Natal_ stocks were produced in C6/36 cells as described (Setoh et al. 2017).

### ZIKV_Natal_ passaging in *Rag1*^-/-^ mice

Adult *Rag1^-/-^* mice (B6.129S7-^Rag1tm1Mom^/J, JAX) bred in-house were infected with ∼10^6^ ffu of ZIKV_Natal_. Mice were weighed three times a week, and when weight loss reached at least 15%, mice were euthanized and blood was taken via heart puncture into MiniCollect serum separation tubes (Greiner Bio-One GmbH, Kremsmunster, Austria). Serum was used for virus titrations by CCID_50_ assays, and 100 µl of serum was passaged into a new *Rag1*^-/-^ mice by intravenous inoculation. Mouse brains were also collected and tissue titres determined by CCID_50_ assays as described (Nakayama et al. 2021a).

### Nanopore sequencing of ZIKV from Rag1^-/-^ mouse serum taken at the end of passage 5

ZIKV RNA was isolated from mouse serum using Nucelospin RNA virus kit (Macherey-Nagel, Düren, Germany), and cDNA was synthesized using ProtoScript II Reverse Transcriptase (New England Biolabs) as per the manufacturer’s instructions. Barcoding polymerase chain reaction (PCR) was performed with ZIKV-specific primers containing the Nanopore universal tail sequences of 5′-TTTCTGTTGGTGCTGATATTGC-3′ for the forward primer and 5′-ACTTGCCTGTCGCTCTATCTTC-3′ for the reverse primer. Primers were designed to amplify approximately 1-kb overlapping amplicons spanning the entire ZIKV genome. PCR using pooled primers (Supplementary Table 2; odd and even numbered primers pooled separately in two reactions to avoid primer interference at the overlapping sequences) was performed using Q5 High-Fidelity 2X Master Mix (New England Biolabs) as per the manufacturer’s instructions with the following thermocycling conditions; 98°C 30 s, 5 cycles of 98°C 10 s, 55°C 30 s, 72°C 1 min, then 25 cycles of 98°C 10 s, 65°C 30 s, 72°C 1 min, and final extension of 72°C 2 min. Amplicons were gel-purified using QIAquick Gel Extraction Kit (QIAGEN). The two amplicon pools for each sample were further pooled and the second round of barcoding PCR was performed using LongAmp Taq 2X Master Mix (New England Biolabs) and Oxford Nanopore barcoding primer sets BC07, BC08, BC09, and BC11, each containing a unique barcode. DNA repair and end-prep using NEBNext FFPE DNA Repair Mix and NEBNext Ultra™ II End Repair/dA-Tailing Module (New England Biolabs) was performed as per the manufacturer’s instructions. Adapter ligation and clean-up were performed using NEBNext Quick Ligation Module (New England Biolabs) as per the manufacturer’s instructions. Sequencing runs were conducted using the MinION Flongle flow cell using MinKNOW software (Oxford Nanopore Technologies, UK) as described (Slonchak et al. 2022). Guppy basecaller (V4.0.11; https://nanoporetech.com/) was used to convert .fast5 files to fastq files. Alignments were undertaken using minimap2 (v2.16) and ZIKV_Natal_ (GenBank: KU527068) as the reference genome. The Integrative Genomics Viewer v2.8.0 (Broad Institute, USA) was used to visualize sequence data.

### Virus stock generation for ZIKV-V153D and ZIKV-N154D

Virus stocks for ZIKV-V153D and ZIKV-N154D were generated by two passages in C6/36 cells. Initially, C6/36 cells were inoculated with serum from viremic *Rag1*^-/-^ mice (taken at euthanasia after passage 5), and were cultured for 5/6 days. Supernatants were then transferred to fresh C6/36 cells and supernatants harvested on 6 dpi, stored at −80°C in aliquots, and then used as virus stocks for subsequent experiments. The glycosylation motif regions from all stocks were sequenced by capillary sequencing.

### Capillary sequencing

To confirm ZIKV RNA sequences in particular regions after virus stock propagation in C6/36 cells or after infection of *Ifnar1*^-/-^ mice, viral RNA was harvested using Nucelospin RNA virus kit (Macherey-Nagel, Düren, Germany), and cDNA was synthesized using ProtoScript II Reverse Transcriptase (New England Biolabs) as per the manufacturer’s instructions. PCR was performed using primers in Supplementary Table 2 that capture the region of interest, or primers to amplify the envelope N-linked glycosylation site region; forward 5ʹ-CTACCTTGACAAGCAATCAG-3ʹ and reverse 5ʹ-CCAACCAGTGCTTGTTATTC-3ʹ. PCR products were gel-purified using Monarch DNA Gel Extraction Kit (New England Biolabs) as per the manufacturer’s instructions and sequences determined by BigDye Terminator v3.1 Sanger sequencing using either the forward or reverse primer. Capillary sequencing was then undertaken using SeqStudio 8 Flex Genetic Analyzer (Applied Biosystems).

### PNGase F treatment and western blot

Vero E6 cells were infected with ZIKV_Natal_, ZIKV-V153D or ZIKV-N154D stock viruses. Cell monolayers were washed with ice-cold PBS before addition of 200 µl trypsin (Sigma) to detach the cells. Cells were collected and centrifuged at 500 g at 4°C for 5 min, and washed with ice-cold PBS and centrifuged again at 500 g, 4°C for 5 min. Cells were resuspended in lysis buffer (50 mM Tris–HCl, 150 mM NaCl, 1 mM EDTA, 1% v/v Triton X-100, pH 7.4) with protease inhibitor cocktail (cOmplete™ Protease Inhibitor Cocktail, Merck Cat# 11,697,498,001) and incubated on ice for 20 min. The lysate was centrifuged at 13,000 g for 20 min and the supernatant was harvested and aliquots stored at −80°C. Twenty µg of protein was treated with PNGase F as per the manufacturer’s instructions (New England Biolabs). Water was used as the mock treatment. The reactions were stopped by adding SDS-PAGE sample buffer [125 mM Tris–HCl, pH 6.8; 4% (v/v) SDS; 20% (v/v) glycerol; 0.004% (w/v) bromphenol blue] and boiling for 10 min. Samples were analysed using 10% Sodium Dodecyl Sulfate–Polyacrylamide Gel Electrophoresis (SDS-PAGE) gel and western blotting using 4G2 (1:100) as the primary antibody incubated at 4°C overnight. Horse radish peroxidase (HRP) goat anti-mouse was use as the secondary antibody (1:2000), and chemiluminescence substrate (SuperSignal™ West Pico PLUS Chemiluminescent Substrate) was used as per the manufacturer’s instructions. Blots were imaged using Biorad ChemiDoc Touch Imaging System, and contrast and brightness adjusted in PowerPoint.

### Growth kinetics in C6/36 or Vero E6 cells

C6/36 and Vero E6 cells were infected in triplicate with the indicated ZIKV stocks at a multiplicity of infection (MOI) of 0.01. C6/36 cells were seeded at 2 × 10^5^ cells in 24-well plate and were incubated at 28°C. Vero E6 cells were seeded at 10^6^ in 6-well plates and were incubated at 37°C. After 1 h, the supernatants were removed and the cells washed in PBS three times before adding fresh culture media (RPMI 1640 supplemented with 10% foetal calf serum). Supernatants were collected at the indicated times and virus titres determined by CCID_50_ assays.

### Human brain organoids; generation, infection, and monitoring

The human-induced pluripotent cells (hiPSCs) utilized in this study were reprogrammed from adult dermal fibroblasts (HDFa, Gibco, C0135C) employing the CytoTune-iPS 2.0 Sendai Reprogramming Kit [Invitrogen, A16518; (Oikari et al. 2020)]. These cells were maintained on human recombinant vitronectin-coated plates (Thermo Fisher Scientific) in StemFlex medium (Thermo Fisher Scientific), according to the manufacturer’s guidelines.

Human cortical brain organoids were generated as outlined (Slonchak et al. 2022; Stewart et al. 2023; Nguyen et al. 2024). hiPSCs were dissociated using StemPro Accutase (Thermo Fisher Scientific) to produce a single-cell suspension. The cells were then plated at a density of 5000 cells per well in an ultra-low-binding 96-well plate (Corning) containing StemFlex medium supplemented with 10 μM ROCK inhibitor Y-27,632 (STEMCELL Technologies, Vancouver, Canada). From Days 1 to 5, the media was replaced daily with StemFlex medium supplemented with 2 μM Dorsomorphine (Abcam) and 10 μM SB-431,542 (STEMCELL Technologies). On Day 5, the medium was replaced with a Neuro-induction medium comprising DMEM/F12 (Thermo Fisher Scientific), 1% N2 Supplement (Thermo Fisher Scientific), 10 μg/ml heparin (STEMCELL Technologies), 1% penicillin/streptomycin (Thermo Fisher Scientific), 1% non-essential amino acids (Thermo Fisher Scientific), 1% Glutamax (Thermo Fisher Scientific), and 10 ng/ml FGF2 (STEMCELL Technologies). On Day 7, the organoids were embedded in Matrigel (Corning), transferred to an ultra-low-binding 24-well plate (Corning) (one organoid per well), and cultured in Neuro-induction medium for an additional three days. On Day 10, the organoids were transitioned to a differentiation medium comprising Neurobasal medium, 1% N2, 2% B27 supplements (Thermo Fisher Scientific), 0.5% penicillin/streptomycin, 1% glutamax, 1% non-essential amino acids, 50 μM 2-mercaptoethanol (Merck), 2.5 μg/ml insulin (Merck), 1% Knockout Serum Replacement (Thermo Fisher Scientific), 10 ng/ml FGF2, and 1 μM CHIR99021 (STEMCELL Technologies). The organoids were cultured for an additional 5 days with media changes performed every other day.

Fifteen-day-old cortical organoids, measuring ∼1 to 1.3 mm in diameter, were infected in quadruplicate with 5 × 10^5^ CCID_50_ of either ZIKV_Natal_, ZIKV-V153D, or ZIKV-N154D stock viruses for 4 h at 37°C in a 5% CO_2_ environment. The organoids were then washed twice with media and cultured in neuro-differentiation media at 37°C, 5% CO_2_ (Nguyen et al. 2020; Stewart et al. 2023). Supernatants were collected at the indicated time points, and viral titres determined by CCID_50_ assays as described (Nakayama et al. 2021a).

Organoids were imaged at various intervals using an EVOS FL microscope (Advanced Microscopy Group), and their 2D image circumference was measured using Image J v1.53 by outlining the organoid periphery (Schneider et al. 2012). At 4 dpi, organoids (*n* = 4 per group) were harvested and formalin-fixed for histology and immunohistochemistry (IHC).

### Immunohistochemistry of human brain organoids

The immunostaining was conducted using the pan-orthoflavivirus 4G4 (anti-NS1) and 4G2 (antienvelope) monoclonal antibodies as described (Hazlewood et al. 2021; Nguyen et al. 2024) using three organoids per virus, with organoids harvested at 4 dpi. Briefly, organoids were embedded in agarose prior to standard paraffin embedding. Sections were stained with 4G4 or 4G2 and HRP-conjugated Perkin Elmer goat antimouse secondary antibody, with signal developed using Vector Nova Red. Sections were lightly counterstained with haematoxylin. Slides were scanned using Aperio AT Turbo (Aperio, Vista, CA USA) and analysed by and Aperio Positive Pixel Count Algorithm (Leica Biosystems) that provided weak positive, positive and strong positive brown staining pixel counts (Nova Red) divided by total count of blue staining pixels (haematoxylin counterstain).

### RNA-Seq and bioinformatics

Total RNA was extracted from organoids using TRIzol (Invitrogen). RNA concentration and quality were measured using TapeStation D1kTapeScreen assay (Agilent). cDNA libraries were generated using Illumina TruSeq Stranded mRNA library prep kit, and were sequenced using an Illumina Nextseq 2000 Sequencing System to produce 75 nt paired-end reads. Sequence reads were aligned to the ZIKA virus genome (KU527068.1) with bowtie2 v2.2.9 (Langmead and Salzberg 2012). Primary proper read alignments to the viral genome were counted using Samtools v1.10 (Li et al. 2009). Sequence reads were aligned to the GRCh38 human reference genome obtained from Gencode, using STAR aligner v2.7.10 (Dobin et al. 2013). Aligned read counts were obtained for annotated genes using RSEM v1.3.1 (Li and Dewey 2011), and differential expression was estimated using DESeq2 v1.32.0 with R statistical software v4.2.0 (Love et al. 2014; R Core Team). We have previously validated our RNA-Seq-derived differential gene expression data using quantitative reverse transcription polymerase chain reaction (RT-qPCR) (Rawle et al. 2021; Bishop et al. 2024; Carolin et al. 2024).

Significantly differentially expressed genes (DEGs) were analysed using Ingenuity Pathway Analysis v107193442 (QIAGEN). Gene set enrichment analyses (GSEA) were performed using GSEA v4.3.2 with gene sets from the Molecular Signatures database v2023 (Subramanian et al. 2005) and the All gene list ranked by fold change.

For single nucleotide variant (SNV) detection, viral read alignments were preprocessed to re-align indels, mark duplicates, and recalibrate basecall quality scores, using lofreq v2.1.5 (Wilm et al. 2012) and the Genome Analysis Toolkit v4.2.4.1 (Van Der Auwera and O’connor 2020). Variants were called using Lofreq with an intra-host allele frequency cutoff of greater than 0.15 and a *P*-value cutoff of less than .05. Intra-host allele frequencies were averaged across replicates and read-depth per SNV was summed across replicates.

### Infection of *Ifnar1*^-/-^ mice

Female *Ifnar1*^-/-^ mice (12- to 13-week-old) (originally provided by Prof. P. Hertzog, Monash University, Melbourne, VIC, Australia; Swann et al. 2007) on a C57BL/6 J background (Rawle et al. 2022) were bred in-house and were infected intraperitoneally with 10^3.2^ CCID_50_ of ZIKV_Natal_, ZIKV-V153D, or ZIKV-N154D virus stocks that had been generated in C6/36 cells. Mice were weighed daily and bled via the tail vein for viremia determinations using CCID_50_ assays.

Serum neutralization titres were determined by incubating 50 µl of heat-inactivated mouse serum (three-fold serial dilutions starting at a 1 in 10 dilution in RPMI 1640 and 2% foetal calf serum) and incubation in duplicate with 1000 CCID_50_ of virus for 3 h (in 50 µl) in a flat well 96-well plate (final lowest serum dilution 1 in 20). Vero E6 cells (Sigma, ECACC Vero C1008) were then added in 100 µl (10^4^/well). After 6 days, overt cytopathic effects (CPEs) were observed by inverted phase light microscopy. The neutralization titre is presented as the highest dilution of serum (mean of duplicates) providing protection from overt CPE.

### Statistics

The *t*-test (unequal variance) was used (in Microsoft Excel 2016, Data Analysis tool) when the difference in variances was <4, skewness was > −2 and kurtosis was <2. Otherwise, the nonparametric Kolmogorov–Smirnov Exact test was used in GraphPad Prism 8.2.1.

## Results

### Passage of ZIKV_Natal_ in *Rag1*^-/-^ mice

**Figure 1.**
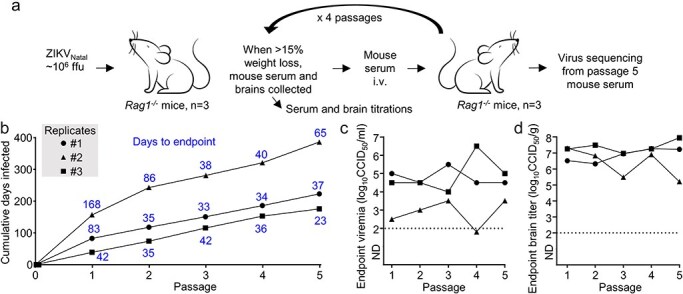
Passage of ZIKV_Natal_ in *Rag1*^-/-^ mice. (a) Experimental design schematic. Three *Rag1*^-/-^ mice were initially infected with ZIKV_Natal_, when weight loss reached >15%, mice were euthanized and serum passaged to new *Rag1*^-/-^ mice; repeated five times in three replicate passage series (*n* = 15 mice in total). (b) For each of the three replicate passage series, the cumulative number of days that the virus was present/replicated in *Rag1*^-/-^ mice. Each point represents the cumulative number of days at which a mouse was euthanized and the serum from that mouse was passaged to the next mouse. Blue numbers; for each of the three replicate passage series, the number of days between virus inoculation and when weight loss reached >15%, requiring the mice to be euthanized is provided. (c) For each of the three replicate passage series, the serum viremia for each mouse at euthanasia. Limit of detection ∼2 log_10_CCID_50_/ml. (d) For each of the three replicate passage series, the brain virus tissue titre for each mouse at euthanasia.

ZIKV_Natal_ was used to infect *Rag1*^-/-^ mice, which have no mature B or T lymphocytes, with viremia and weight loss subsequently monitored. The animals were euthanized when the ethically defined end point of ≥15% body weight loss was reached. Serum from euthanized mice was then passaged into new *Rag1*^-/-^ mice. This process was repeated for five passages in *Rag1*^-/-^ mice, with passaging conducted in three replicate series comprising three independent parallel mouse-to-mouse transfer series (five mice per series) (Fig. 1a). The cumulative amount of time that *Rag1*^-/-^ mice were infected with ZIKV_Natal_ ranged from 180 days (Replicate #3 series) to 400 days (Replicate #2 series) (Fig. 1b). The time to the weight-loss endpoint (≥15%) and euthanasia tended to decrease after 1–2 passages and stabilized at approximately 30–40 days for the remaining three passages in all three replicates (Fig. 1b, blue numbers; Supplementary Fig. 1a).

The serum titres in each *Rag1*^-/-^ mouse over the initial 2–6 dpi for each of the Replicate series were relatively low for the first 1–2 passages, thereafter (passages 3–5) they increased and remained at ∼2 to 3 log_10_CCID_50_/g (Supplementary  Fig. 1b). The serum titres at euthanasia did not show a clear trend with passage number (Fig. 1d), with arboviral viremias in *Rag1^-/-^* mice tending towards a steady state level after 1–2 weeks (Poo et al. 2014). However, one mouse had an undetectable viremia at passage 4 (limit of detection was ∼2 log_10_CCID_50_/ml); nevertheless, serum transfer successfully established a detectable viremia at passage 5 (Fig. 1d). The age of each adult mouse used in the passaging is provided in Supplementary  Fig. 1c; no significant correlations associated with mouse age emerged (Nakayama et al. 2021a).

Brain ZIKV_Natal_ titres at euthanasia were consistently between 5 and 8 log_10_CCID_50_/g (Fig. 1e), with no significant increases in brain titres at euthanasia associated with serial passage. Brain infection and associated weight loss in ZIKV-infected *Rag1*^-/-^ mice have been described previously, with infection largely limited to brain in this mouse model (Hayashida et al. 2019). Brain infection may be causally associated with weight loss (Ajithkumar et al., 1998; Lazear et al. 2016; Stewart et al. 2023), and as mice were euthanized at similar levels of weight loss (≥15%), such consistent levels of brain infection might be anticipated. *Rag1*^-/-^ mice have intact type I IFN responses, with ZIKV NS5 able to inhibit human, but not mouse, type I IFN responses (Grant et al. 2016a; Slonchak et al. 2022). Neurons are often unable effectively to mount such responses (Delhaye et al. 2006), with these cells a primary target of ZIKV infection in *Rag1*^-/-^ mice (Hayashida et al. 2019). Such neurotropism was retained after passaging, with passage 5 virus infecting *Rag1*^-/-^ brain cells with neuronal morphology (Stewart et al. 2023; Nguyen et al. 2024) (Supplementary  Fig. 2).

**Figure 2.**
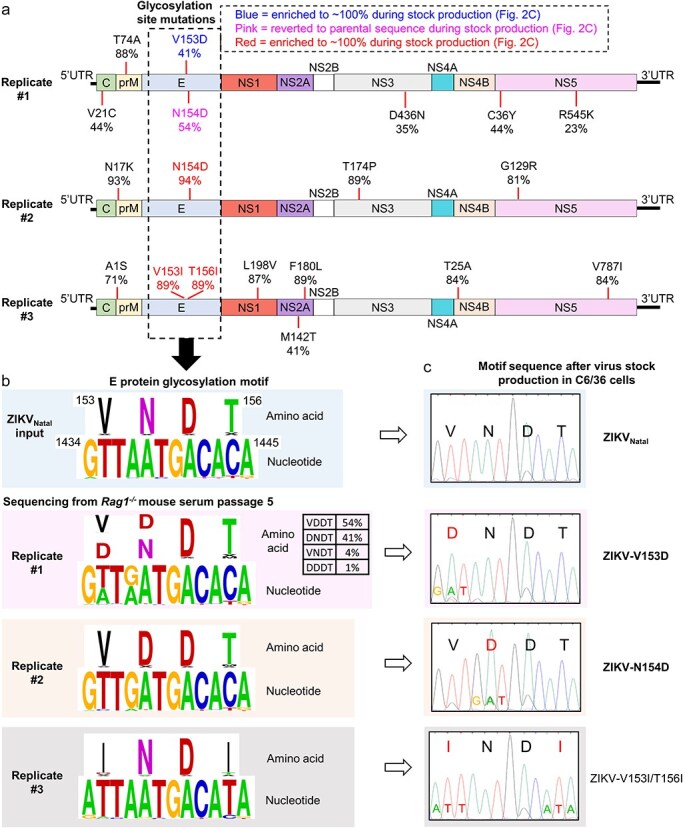
Sequences of ZIKV_Natal_ after passage in *Rag1*^-/-^ mice. (a) Amino acid changes in the ZIKV_Natal_ sequences of viruses in serum of *Rag1*^-/-^ mice after passage 5 for each of the three replicate passage series. Sequences were obtained by Nanopore sequencing. Reference genome KU527068.1. (b) Amino acid and nucleotide sequences of the envelope N-linked glycosylation motif of the parental ZIKV_Natal_ and the viruses described in (a). Amino acid and nucleotide numbering from KU527068.1. (c) Envelope N-linked glycosylation motif sequences after generation of virus stocks by two passages in C6/36 cells. Sequences were obtained by capillary sequencing of PCR products (Supplementary Fig. 2a and b).

### ZIKV passage in *Rag1*^-/-^ mice selects for envelope N154-linked glycosylation motif mutants

After five passages in *Rag1*^-/-^ mice, the virus in the serum from euthanized mice (end of passage 5) for each of the three replicate series was sequenced using Nanopore sequencing. Sequences were aligned to the ZIKV_Natal_ reference genome and nucleotide changes ≥ 15% were manually curated using Integrated Genomics Viewer (IGV) (Supplementary Table 3). Across the three replicates, there were 42 total nucleotide mutations detected resulting in approximately 50% synonymous and 50% nonsynonymous amino acid mutations (Supplementary Table 3). The selected amino acid changes are shown in Fig. 2a. In all three independent replicates, the envelope N-linked glycosylation motif was disrupted, with this involving selection of three different patterns of nucleotide and amino acid changes (Fig. 2b). The ZIKV_Natal_ N-linked envelope glycosylation motif comprises the amino acids VNDT at positions 153–156, although inclusion of V^153^ has only been proposed (Gong et al. 2018; Frumence et al. 2019; Gallichotte et al. 2023). The dominant sequence changes in this motif were Replicate #1 VDDT (54%) and DNDT (41%), Replicate #2 VDDT, and Replicate #3 INDI (Fig. 2b).

Viruses from *Rag1*^-/-^ mouse serum (end of passage 5) were passaged twice in C6/36 cells to produce virus stocks, with capillary sequencing of the glycosylation site illustrating that disruptions in the N-linked glycosylation motif were retained in the virus stocks (Fig. 2c; Supplementary  Fig. 3a and b). Although Replicate #1 presented a mixed population in *Rag1*^-/-^ mouse serum (Fig. 2a and b), the DNDT sequence emerged as dominant after passage in C6/36 cells (Fig. 2c, ZIKV-V153D), with T74A and D436N also retained (Supplementary  Fig. 3c).

**Figure 3.**
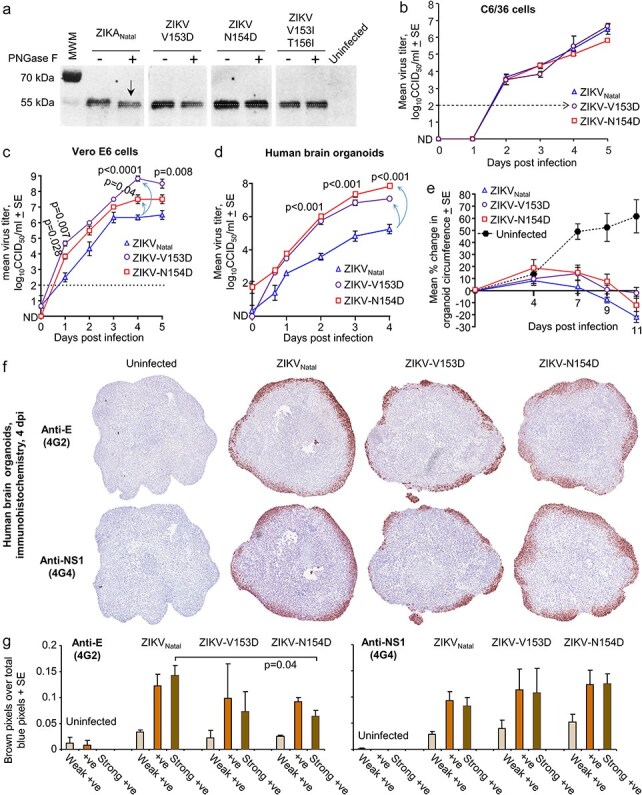
Glycosylation and replication of glycosylation motif mutants. The following experiments were conducted using C6/36-derived virus stocks. (a) The indicated viruses were used to infect Vero E6 cells and cell lysates (4 dpi) were treated with PNGase F and analysed by western blotting using the 4G2 anti-E monoclonal antibody. The reduction in molecular weight is evident for ZIKV_Natal_ (white arrow). MWM—molecular weight markers. (b) Growth kinetics of the indicated (C6/36 cell-derived) virus stocks in C6/36 cells. Limit of detection ∼2 log_10_CCID_50_/ml. (c) Growth kinetics of the indicated (C6/36 cell-derived) virus stocks in Vero E6 cells. At 4 dpi the mean titre for ZIKV-V153D was ∼2.5 logs higher, and the mean titre for ZIKV-N154D was ∼1 log higher, than ZIKV_Natal_ (light blue arrows). Statistics by *t*-test, all vs. ZIKV_Natal_. (d) Growth kinetics of the indicated (C6/36 cell-derived) virus stocks in 15 day old human cortical brain organoids (mean titres were derived from four replicates for each virus). At 4 dpi the mean titres for ZIKV-V153D were ∼1.8 logs higher, and for ZIKV-N154D the mean titres were ∼2.6 logs higher, than ZIKV_Natal_ (light blue arrows). Statistics by *t*-test vs. ZIKV_Natal_. (e) Mean percentage change in organoid circumference at 11 dpi vs. 0 dpi for each organoid (*n* = 4 organoids per group). (f) IHC of brain organoids at 4 dpi using pan-orthoflavivirus anti-NS1 (4G4) and anti-E (4G2) monoclonal antibodies. The diameter of the uninfected 15 day old organoids was ∼1 to 1.3 mm. (g) Aperio pixel count analysis of antibody staining (weak positive—light brown), positive (brown), strong positive (dark brown), divided by total blue staining (haematoxylin counterstain). Means shown for *n* = 3. Statistics by *t*-test.

In summary, during serial passage of ZIKV_Natal_ in *Rag1*^-/-^ mice, disruptions of the envelope N-linked glycosylation motif were identified in all three independent replicates. Each disruption was distinct and glycosylation motif disruptions were retained after generation of viral stocks in C6/36 cells (Fig. 2c; Supplementary  Fig. 3a and b). These viral stocks were used in subsequent experiments and the viruses referred to as ZIKV-V153D, ZIKV-N154D, and ZIKV-V153I/T156I (Fig. 2c).

### PNGase treatment to assess N-linked glycosylation of ZIKV envelope proteins

To confirm that the aforementioned mutations disrupt the N-linked glycosylation of the envelope protein, C6/36-derived viral stocks were used to infect Vero E6 cells, and at 4 dpi, cells were harvested and cell lysates treated with PNGase F and analysed by western blotting using the 4G2 anti-E monoclonal antibody. The reduction in molecular weight was evident for envelope from ZIKV_Natal_ (Fig. 3a, white arrow). No reductions in the molecular weight of envelope from ZIKV-V153D, ZIKV-N154D, or ZIKV-V153I/T156I were evident after PNGase F treatment (Fig. 3a, white dashed line). These results illustrate that the envelope proteins from these latter three viruses have no dectable levels of N-linked glycosylation.

Several reports have proposed that the glycosylation motif might include a V (Gong et al. 2018; Frumence et al. 2019; Gallichotte et al. 2023); thus VNDT in the current setting (Fig. 2b). The result for ZIKV-V153D (Fig. 3a) would support this contention, or at least argues that V^153^ promotes efficient glycosylation and/or that the D^153^ substitution interferes with efficient glycosylation in this setting.

### ZIKV N-linked glycosylation mutants show increased replication in Vero E6 cells and human brain organoids

Replication kinetics of C6/36-derived virus stocks were assessed in C6/36 cells. Growth of ZIKV-V153D, ZIKV-N154D, and ZIKV_Natal_ were very similar in C6/36 cells (Fig. 3b). ZIKV-V153I/T156I replicated very poorly in C6/36 cells (Supplementary  Fig. 4), and was thus not used in further experiments. In Vero E6 cells, ZIKV-V153D and ZIKV-N154D both replicated to significantly higher titres (∼2.5 and 1 logs, respectively) when compared to the parental ZIKV_Natal_ (Fig. 3c).

**Figure 4.**
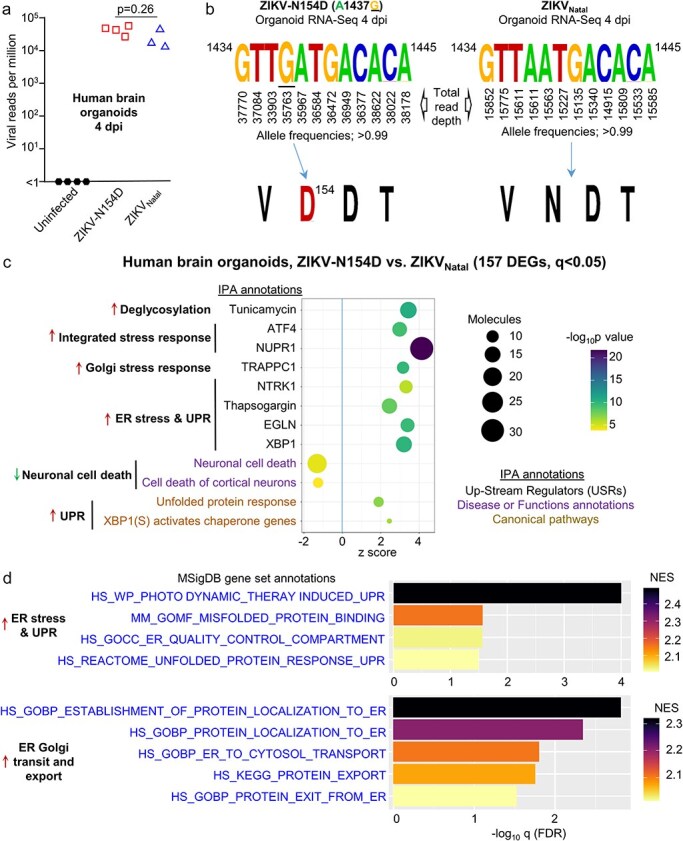
Brain organoid RNA-Seq. (a) Viral read counts per million at 4 dpi for organoids infected with the indicated virus. Statistic by *t*-test (not significant). (b) Left—retention of N154D substitution for the ZIKV-N154D after two passages in C6/36 cells and 4 days growth in organoids. Right—no nucleotide changes were identified after 4 days growth of ZIKV_Natal_ in organoids. The read depth at each position is derived from the combined data from all organoids infected with the indicated virus. (c) Ingenuity Pathway Analyses (IPA) of DEGs identified by RNA-Seq data comparing ZIKV-N154D with ZIKV_Natal_ infected organoids at 4 dpi. Selected annotations are shown and are grouped into themes (bold text), with arrows indicating up-regulation (red) or down-regulation (green). (d) GSEA analysis of ranked All gene list using MSigBD gene sets, selected annotations are shown as for C. Full list of genes and bioinformatic analyses are provided in Supplementary Table 4.

Human brain organoids have been used extensively to study ZIKV infection of neuronal cells (Sutarjono 2019; Slonchak et al. 2022). Early (15 day old) human cortical brain organoids were infected with C6/36-derived stocks of ZIKV_Natal_, ZIKV-V153D, and ZIKV-N154D, and viral titres in culture supernatants determined over time. Consistent with data from Vero E6 cells (Fig. 3c), ZIKV-V153D and ZIKV-N154D both replicated to significantly higher titres (∼1.8 and 2.6 logs, respectively, at 4 dpi) compared to ZIKV_Natal_ in the human brain organoids (Fig. 3d, blue arrows). Curiously, reductions in organoid circumferences, driven by ZIKV CPE (Slonchak et al. 2022), were not significantly higher for ZIKV-V153D or ZIKV-N154D, when compared with ZIKV_Natal_ (Fig. 3e). Furthermore, IHC of organoids at 4 dpi did not indicate significantly increased levels of envelope (4G2) or NS1 (4G4) protein staining for ZIKV-V153D or ZIKV-N154D-infected organoids, when compared with ZIKV_Natal_-infected organoids (Fig. 3f and g), despite the log-higher levels of virus replication at 4 dpi (Fig. 3d). Pixel count analysis of strong brown 4G2 staining was actually slightly, but significantly, lower for ZIKV-N154D-infected organoids (Fig. 3g, *P* = 0.04).

In summary, ZIKV-V153D and ZIKV-N154D showed significantly increased replication (up to ∼2.6 logs) in Vero E6 cells and human brain organoids when compared with ZIKV_Natal._ IHC and size analyses of infected human brain organoids indicated that the log increases in replication were not associated with infection of more cells, increases in the levels of viral proteins, or substantially increased levels of CPE.

### RNA-Seq suggests more robust induction of the unfolded protein response by ZIKV-N154D

To gain insights into the processes that might underpin the increased replication of the glycan mutants (Fig. 3d), ZIKV-N154D-infected human cortical organoids were compared with ZIKV_Natal_-infected organoids at 4 dpi by RNA-Seq. ZIKV-N154D was chosen as the N154D mutation is unequivocally associated with an absence of N-linked glycosylation, as the glycan is attached to the side chain of N^154^.

The number of viral RNA reads was not significantly different for ZIKV-N154D vs. ZIKV_Natal_ (Fig. 4a), despite the log increases in the levels of infectious virus in the supernatants of these organoid cultures (Fig. 3d). Taken together, these data suggested that the steady-state viral RNA levels in the infected cells were largely unchanged, but that mature virion egress was substantially accelerated. The viral RNA-Seq reads (Supplementary Fig. 5) illustrated that the N154D substitution for ZIKV-N154D was retained at 4 dpi, and that the glycosylation motif for ZIKV_Natal_ was also unchanged (Fig. 4b).

Analyses of the host cell transcriptome provided 157 DEGs (q < 0.05) for ZIKV-N154D vs. ZIKV_Natal_ (Supplementary Fig. 6, Supplementary Table 4), with these DEGs then analyzed by Ingenuity Pathway Analyses (IPA). Tunicamycin was identified as significant IPA upstream regulator (Fig. 4c), perhaps expected as this drug inhibits N-linked glycosylation. Annotations associated with the Golgi stress response, TRAPPC1 (Passemard et al. 2019) and the integrated stress response, NUPR1 (Liu and Costa 2022) and ATF4 (Neill and Masson 2023), were also identified. Several IPA annotations indicated an increase in ER stress and the unfolded protein response (UPR). These included XBP1 (Tan et al. 2018; Kolpikova et al. 2020; Breitkopf et al. 2021) and Thapsogargin (Kim et al. 2022), with NTRK1 (Jiao et al. 2023) and EGLN (Ajoolabady et al. 2021) also associated with the UPR (Fig. 4c). IPA Diseases and Functions annotations also indicated lower levels of neuronal cell death in ZIKV-N154D-infected organoids when compared with ZIKV_Natal_-infected organoids (Fig. 4c, Supplementary Table 4). This is consistent with a marginal trend towards less CPE for ZIKV-N154D vs. ZIKV_Natal_ as indicated by organoid size, although this did not reach significance (Fig. 3e).

GSEAs were undertaken using the ranked ‘All gene’ list (Supplementary Table 4) and gene sets from the Molecular Signatures Database (MSigDB) (Liberzon et al. 2015). A series of annotation associated with ER stress/UPR were again identified (Fig. 4d), consistent with the IPA data. A series of MSigDB annotations also suggested increased protein traffic through the ER and Golgi, and out of the cell (Fig. 4d, ER Golgi transit and export). Such activities would be consistent with a successful UPR, which leads to upregulation of secretory pathway chaperones and foldases that reduce the nascent protein load in the ER (Read and Schröder 2021) by increasing protein export (Shaheen 2018; Raschmanová et al. 2021; Lin et al. 2023). A successful UPR can also mitigate against cell death (Read and Schröder 2021).

In summary, the RNA-Seq data argue that the glycan mutant, ZIKV-N154D, induces a more robust and successful UPR than the glycosylated parent ZIKV_Natal_, which results in accelerated transit of the viral envelope protein, and the assembling virion, through the ER and Golgi and out of the cell. Such activity would be consistent (Raschmanová et al. 2021; Lin et al. 2023) with the higher viral titres in the supernatant (Fig. 3d), and the lack of significant increases in viral protein staining as measured by IHC (Fig. 3f and g).

### ZIKV-V153D and ZIKV-N154D replicate faster in *Ifnar1*^-/-^ mice

**Figure 5.**
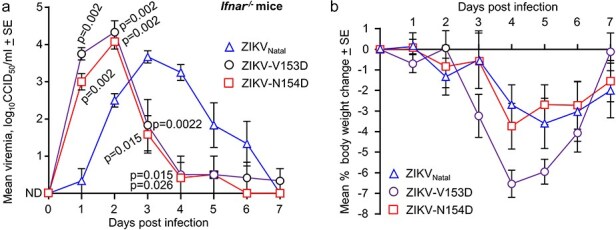
In mice ZIKV-V153D and ZIKV-N154D showed more rapid onset, but shorter viremic periods than ZIKV_Natal_. (a) Mean viremias in *Ifnar^-/-^* mice (*n* = 6 per group) after infection with ZIKV_Natal_, ZIKV-V153D, or ZIKV-N154D. The *P* values displayed on the graph represent Kolmogorov–Smirnov exact tests, and are all relative to ZIKV_Natal_. At 1 dpi the mean titre for ZIKV-V153D was 0.75 logs higher than for ZIKV-N154D, *P* = .026, *t*-test (this *P* value is not displayed on the graph). The peak mean titres for ZIKV-V153D and ZIKV-N154D (2 dpi) were ∼0.7 and ∼0.4 logs higher, respectively, than the mean peak titre for ZIKV_Natal_ (3 dpi), although these differences did not reach significance. ND—not detected in all mice; limit of detection per mouse was ∼2 log_10_CCID_50_/ml. (b) Mean percentage change in mouse body weight for the mice described in A.

To determine whether the increased replication of ZIKV-V153D and ZIKV-N154D in Vero E6 cells and in human brain organoids is recapitulated *in vivo*, these viruses were used to infect *Ifnar1^-/-^* mice. ZIKV infection in this mouse model is more disseminated than in *Rag1*^-/-^ mice and recapitulates aspects of human pathogenesis (Tripathi et al. 2017; Hayashida et al. 2019). Infection with ZIKV-V153D and ZIKV-N154D resulted in the viremia peaking at 1–2 dpi and falling substantially by 4 dpi, whereas the viremia for ZIKV_Natal_ peaked at 3–4 dpi, dropping by 6–7 dpi (Fig. 5a). The glycan mutant viruses thus initially replicated faster, but the viremia was more rapidly curtailed, with the viremic periods thus emerging to be shorter for the glycan mutants. Specifically, ZIKV-V153D and ZIKV-N154D had mean viremia titres of >1 log_10_CCID_50_/ml for ∼3.2 days, whereas ZIKV_Natal_ infection showed mean viremia titres of >1 log_10_CCID_50_/ml for ∼5 days.

The loss of infectious ZIKV titres from serum generally coincides with the development of neutralizing antibody responses (Diamond et al. 2003; Singh et al. 2022), with high doses of antigen often resulting in early induction of both IgM and IgG responses in mice (Black and Inchley 1974; Lucas et al. 2018). The more rapid curtailment of the viremias in ZIKV-V153D- and ZIKV-N154D-infected mice (Fig. 5a) therefore suggests enhanced virus neutralization in the mice infected with these glycan mutants. Comparing neutralizing antibody activities using sera taken 7 dpi from the mice shown in Fig. 5a provides support for this contention (Supplementary Fig. 8).

Spleen was harvested from a subgroup of mice at 3 dpi, and the N-linked glycosylation site was analysed by capillary sequencing. The results illustrated that no reversion to intact glycosylation motifs had occurred by 3 dpi for ZIKV-V153D and ZIKV-N154D (Supplementary Fig. 7). Although restoration of the glycosylation motif was observed for ZIKV-MR766-NIID (Nakayama et al. 2021a), it should be noted that ZIKV-MR766-NIID produces a peak viremia of ∼6 log_10_CCID_50_/ml in *Ifnar1^-/-^* mice (Nakayama et al. 2021a), ∼2 logs higher than those seen herein for ZIKV_Natal_ (Fig. 5a). Less replication provides less opportunity for mutation, selection and thus reversion.

Monitoring of body weight post infection indicated clear reductions in weight, although no mouse reached the ethically defined end point of >15% body weight loss (Fig. 5b). Infection with ZIKV-V153D virus was associated with some increased weight loss compared to the other viruses (Fig. 5b), perhaps due to the slightly higher mean viremia (Fig. 5a). Importantly, infection with the glycosylated parental ZIKV_Natal_ was not associated with increased levels of weight loss or overt pathology when compared to the glycan mutants (Fig. 5b). These results do not support the contention that envelope N-linked glycosylation plays a substantial role in promoting pathology (Feng et al. 2022), at least in this ZIKV mouse model.

## Discussion

Herein we used evolution in *Rag1*^-/-^ mice to select for envelope N-linked glycosylation motif mutants, rather than using genetic engineering to generate such mutants. This approach had the advantage of naturally selecting motif mutations that imparted enhanced replication fitness. This contrasts with approaches that engineer specific amino acid substitutions (Supplementary Table 1), whose impacts (separate from glycan loss) on the envelope protein and virus replication are largely unknown. Two glycan mutants selected in *Rag1*^-/-^ mice, ZIKV-V153D and ZIKV-N154D, showed increased replication kinetics in Vero E6 cells and in human brain organoids (up to ∼2.6 logs higher), when compared with the parental glycosylated ZIKV_Natal_. These observations are consistent with studies on other orthoflaviviruses, which show increased replication of envelope N-linked glycan mutants in brains when using animals models where antibodies are largely absent due to young age (Kawano et al. 1993; Kariwa et al. 2013; Guo et al., 2021), immunodeficiency (Halevy et al. 1994; Chambers et al. 2008), or intracranial delivery (Cheng et al. 2022; Fang et al. 2022).

Our observations are not consistent with the contention that envelope N-linked glycosylation of ZIKV (and other orthoflaviviruses) is required for proper folding, processing, stability, maturation and/or transport of envelope, and the assembling virion, into the extracellular milieu (Courageot et al. 2000; Mossenta et al. 2017; Gwon et al. 2020; Ishida et al. 2023; Bishop et al. 2024; Pandey et al. 2025). However, such findings may often arise from comparisons of wild-type virus with an engineered glycosylation-motif mutant, where the envelope amino acid sequence changes may impart unfavourable properties, with the loss of the glycan residue a secondary issue. For instance, the N154A substitution (Annamalai et al. 2017; Liang et al. 2018; Liu et al. 2021; Xu et al. 2023) may itself promote instability, insolubility and/or degradation (Ishida et al. 2023) and/or toxicity (Steiner et al. 2023). The data presented herein argue that the best mutations for disrupting the glycosylation motif, without introducing a detrimental amino acid change is N154D or V153D. Although these substitutions have previously been identified (Liu et al. 2022; Gallichotte et al. 2023), to the best of our knowledge, N154D or V153D mutants have not been used to study the role of glycosylation (Supplementary Table 1).

ZIKV would not likely have evolved its envelope N-linked glycosylation in order to improve virus replication, as D in position 153 or 154 achieves this goal with substantially greater efficiency. A critical role for lectin-assisted infection processes (Carbaugh et al. 2020; Contreras et al. 2024) is also not supported by our data. Instead, our data are consistent with the established contention that such envelope glycans play an important role in the evasion of neutralizing antibody responses. This concept has been proposed for ZIKV, based on the proximity of the glycan moieties to the fusion loop (Sirohi et al. 2016; Frumence et al. 2019) (Supplementary Fig. 9a) and was recently supported by experimental data (Contreras et al. 2024). Antibodies specific for fusion loop regions are viewed as having highly effective anti-orthoflavivirus neutralizing activity (Kotaki et al. 2021; França et al. 2022; Fan et al. 2024). Glycan-mediated evasion of neutralizing antibodies has also been reported for other viruses (Karlsson Hedestam et al. 2008; Sommerstein et al. 2015; Lavie et al. 2018). The concept is also clearly illustrated by the technique known as ‘glycan masking’ or ‘glycan shielding’ whereby glycans are engineered into specific regions in the immunogen to direct antibodies response away from those regions (Shinnakasu et al. 2021; Martina et al. 2023; Zhang et al. 2023a). A key consequence of an arbovirus delaying/evading early effective neutralizing antibody responses is to extend the viremic period. Our data support the view that N-linked envelope glycosylation can reduce antibody neutralization activity (Supplementary Fig. 8), thereby extending the ZIKV viremic period (Fig. 5a). Although not a selection pressure in play in our mouse model, lengthening the viremic period would increase the probability of virus transmission from the mammalian host to mosquito vectors, an important factor in arbovirus spread and evolutionary selection (Armstrong et al. 2020; Fesce et al. 2023).

So why would our glycan mutants replicate better? Firstly, RNA-Seq analysis of ZIKV-N154D vs. ZIKV_Natal_ infection of organoids indicated that ZIKV-N154D infection led to increased stimulation of the UPR. Importantly, this was not associated with increased neuron cell death signatures, which were actually slightly reduced. That infection with orthoflaviviruses (including ZIKV) induces the UPR is well documented, with the UPR generally viewed as promoting viral replication (Lewy et al. 2017; Tan et al. 2018; Kolpikova et al. 2020; Breitkopf et al. 2021;Viettri et al. 2021;Yeh et al., 2023). A UPR-mediated upregulation of *inter alia* secretory pathway chaperones and foldases benefits viral replication, as movement of viral structural proteins and assembling virions through the ER and Golgi and out of the cell is accelerated (Shaheen 2018; Raschmanová et al. 2021; Lin et al. 2023). The UPR can lead to apoptosis if the ER stress is not resolved, with UPR-driven apoptosis widely studied (Kapuy 2024). However, if stress is resolved, with unfolded proteins cleared from the ER, then cell death can be avoided or delayed (Read and Schröder 2021; Kovaleva et al. 2023). In viral infection settings, prolonging host cell survival would clearly promote virus replication (Ambrose and Mackenzie 2011; Prasad and Greber 2021). A second, likely related, reason why ZIKV-N154D and ZIKV-V153D replicate better may be associated with the high metabolic and kinetic costs of glycosylation (Mariño et al. 2010; Del Val et al. 2016). Glycosylation involves numerous sequential and competitive steps in an assembly line-like process (Breitling and Aebi 2013) that requires both energy and time to complete (Scheper et al. 2023). During orthoflavivirus replication, the ER is extensively reorganized to prioritize synthesis and secretion of glycosylated orthoflavivirus structural proteins (Aktepe and Mackenzie 2018; Bishop et al. 2024). A fitness cost for N-linked envelope glycosylation is consistent with the selection of the three glycan mutants during passage in *Rag1*^-/-^ mice. The bioinformatic analysis suggests accelerated ER/Golgi transit and viral egress for ZIKV-N154D. Arguably this would (i) be easier to achieve without the requirement for glycosylation and (ii) be consistent with a more effective UPR. Thirdly, the V153D and N154D substitutions may not impart unfavourable properties, unlike, for instance, the N154A substitution, which imparts instability, insolubility and degradation (Ishida et al. 2023) and toxicity (Steiner et al. 2023). Perhaps important is that both N154D and V153D preserve (via the aspartic acid residues) a negative charge on the surface of the envelope protein in the location of the glycan loop (Supplementary Fig. 9B). Glycosylated ZIKVs also have a negative charge in this location, provided by the sialic acid residues of the glycan loop (Routhu et al. 2019). Such surface negative charge is lost for the N154A substitution (Supplementary Fig. 9b). One might thus speculate that the N154D and V153D substitutions can replace the glycan moiety, without imparting unfavourable properties, by preserving negative charge at the glycosylation motif site (Qing et al. 2022). A related observation was reported for *Saccharomyces cerevisiae* proteins expressed under nutrient-deficient conditions, which resulted in loss of N-glycosylation and the introduction of charged amino acids at the N-glycosylation sites. These latter amino acid substitutions increased protein stability and activity (Tan et al. 2014).

Curiously some orthoflaviviruses appear not to carry an envelope N-linked glycan. For instance, Kunjin virus isolates are often not glycosylated; however, this may be due to passage history, rather than a feature of the virus in naturally infected mammalian hosts (Adams et al. 1995). For instance, during an outbreak of Kunjin encephalitis in horses in 2011 in Australia, which resulted in ≈10-15% mortality (Prow 2013), a primary isolate was obtained that did have an N-linked envelope glycan (Frost et al. 2012). Loss of the envelope N-linked glycosylation motif associated with *in vitro* culture has also been reported for a number of other orthoflaviviruses (Lee et al. 1997; Shirato et al. 2004; Haddow et al. 2012). Thus both passage in *Rag1*^-/-^ mice and passage *in vitro* can select viruses lacking N-linked envelope glycosylation, with both selections occurring under conditions that lack neutralizing antibodies.

Our paper has a number of limitations. Although we have assessed replication in C6/36 cells, we have not assessed fitness in mosquitoes, leaving open the question of whether ZIKV envelope protein glycosylation is required for efficient viral dissemination from the mosquito midgut to the saliva (Moudy et al. 2009; Setoh et al. 2017; Wen et al. 2018). Also unclear is why ZIKV-V153I/T156I replicated so poorly in C6/36 cells (Supplementary  Fig. 4). Retention of a positively charged patch at the glycan loop site (Supplementary Fig. 9) would not appear to be important for replication in C6/36 cells (Fig. 3b), suggesting V153I/T156I mutations impart a distinct disadvantage in insect cells that does not manifest in *Rag1*^-/-^ mice. That replication in insect and mammalian cells selects for quite distinct sets of substitutions in ZIKV_Natal_ envelope has been shown by deep mutational scanning experiments (Setoh et al. 2019). We are also unable to provide cogent insights into the other substitutions outside the glycosylation motif (Fig. 2a, Supplementary  Figs 3c and 5a), except that they are entirely different for each of the three selected glycan mutants (Fig. 2A), none match amino acids seen in the mouse-adapted ZIKV-MR766 strain (Supplementary Table 3), none are located in envelope, and none, to the best of our knowledge, have been associated with increased viral fitness in mammalian cells. Lastly, we did not expand our studies to other more virulent ZIKV strains, where it would now be interesting to engineer the D substitutions into the envelope glycosylation motifs and determine whether *in vitro* replication in mammalian cells would again be substantially increased. Evolution of glycan mutants in *Rag1*^-/-^ mice may not occur for all viral strains (Gorman et al. 2018), as other virulence factors (Nakayama et al. 2021a; Boytz et al. 2024; Perera et al. 2024) may dominate or confound the process.

In summary, our data argue that the N-linked glycosylation of the ZIKV_Natal_ envelope protein is not essential for supporting envelope folding, processing, assembly, and/or transit through the ER and Golgi in primate cells. If anything, glycosylation slows this process, with the glycan mutants ZIKV-V153D and ZIKV-N154D yielding ∼1 to 2.6 logs more virus. Instead, N-linked glycosylation of envelope was associated with extension of the viremic period, consistent with ‘glycan masking/shielding’ activity (Lavie et al. 2018; Martina et al. 2023; Zhang et al. 2023a) that would delay/avoid the generation and activity of early neutralizing antibody responses. Thus, in the absence of antibodies, the fitness cost of glycosylation can be revealed, whereas in real-world setting where antibody responses are being generated, glycosylation would be selected for, in order to slow development of antibody-based virus neutralization and thereby promote transmission to mosquitoes. Future work might seek to determine whether the results presented herein also apply to the envelope glycosylation of other orthoflaviviruses (Supplementary Table 1), and perhaps also explore whether such D substitutions in the glycosylation motif might improve yield and/or bioactivity of whole-virus or virus-like-particle vaccines and/or therapeutics (Cimica et al. 2021; Lin et al. 2022; Chen et al. 2025).

## Supplementary Material

veaf021_Supp

## Data Availability

All data are provided in the manuscript and accompanying supplementary files. Raw sequencing data (fastq files) generated for this publication have been deposited in the NCBI SRA, BioProject: PRJNA1141163 and are publicly available as of the date of publication.
