# Supplementary material for: Evolution of Zika virus in Rag1-deficient mice selects for unique envelope glycosylation motif mutants that show enhanced replication fitness
Source: Virus Evol. 2025 Apr 11;11(1):veaf021. doi: 10.1093/ve/veaf021 (PMC12024116; doi:10.1093/ve/veaf021)
Supplement: veaf021_Supp [file veaf021_supp.zip › suppl_data/Supplementary Figures_revised.pdf]

A

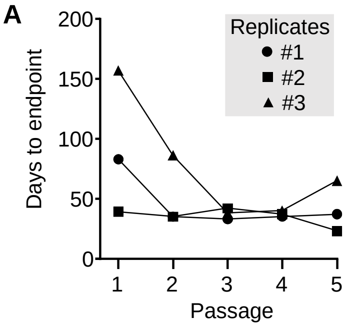

B

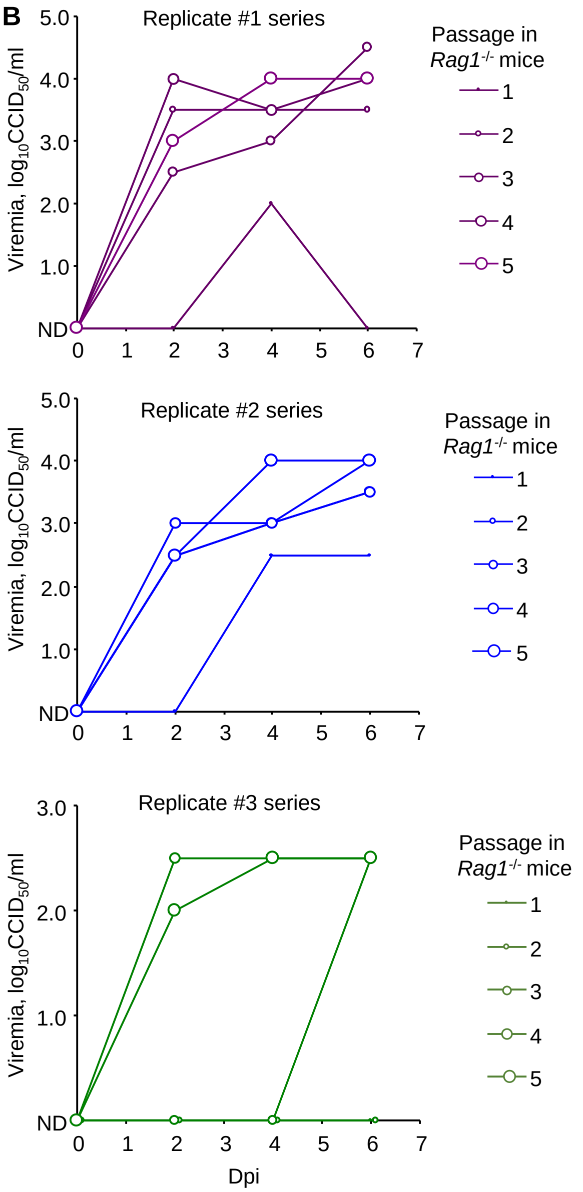

C

Mouse age (weeks)

	Passage				
Replicate	1	2	3	4	5
#1	9	13	19	22	10
#2	9	12	13	21	20
#3	11	21	21	18	6

Supplementary Fig. 1. (A) Blue numbers in Fig. 1B graphed. Each data point represents one mouse. Days to end point represent the number of days between inoculation of virus into the Rag1$^{-/-}$ mouse and the time when that mouse was euthanized after reaching the ethically defined end point of $\geq$15% body weight loss. Serum from that mouse when then passaged into the next mouse. (B) Viremia during the first 6 days of infection of each of the *Rag1*$^{-/-}$ mice for each of the 5 passages and each of the 3 replicates. Each line represents course of viremia in a single mouse. ND – not detected (limit of detection ≈ 2 $\log_{10}$CCID$_{50}$/ml). (C) Age of each Rag1$^{-/-}$ mouse at the time of virus inoculation. No significant correlations associated with mouse age emerged; lack of correlation of viral parameters and age of adult mice were also reported for the *Ifnar1*$^{-/-}$ mouse model of ZIKV infection (Nakayama et al., 2021a).

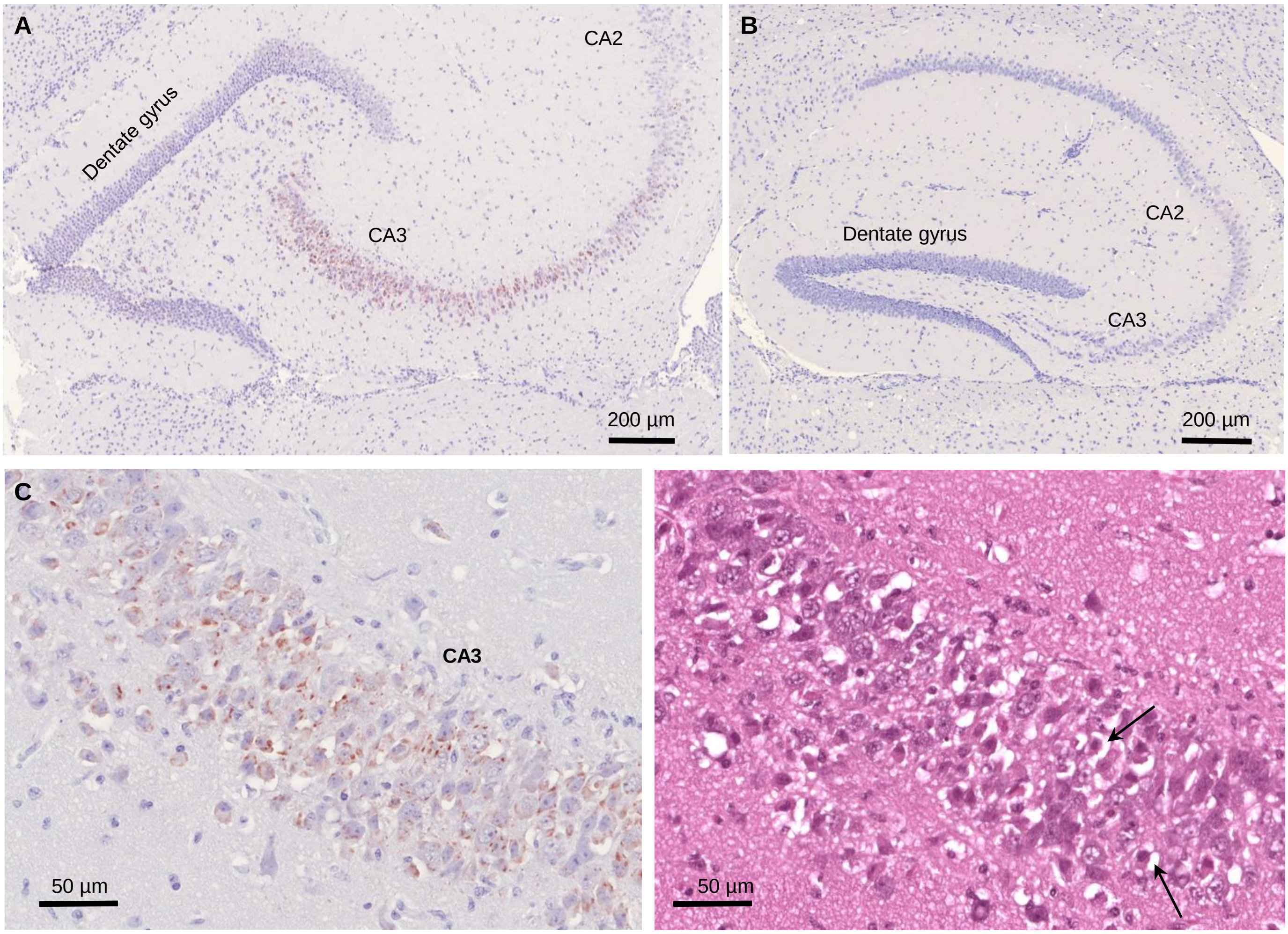

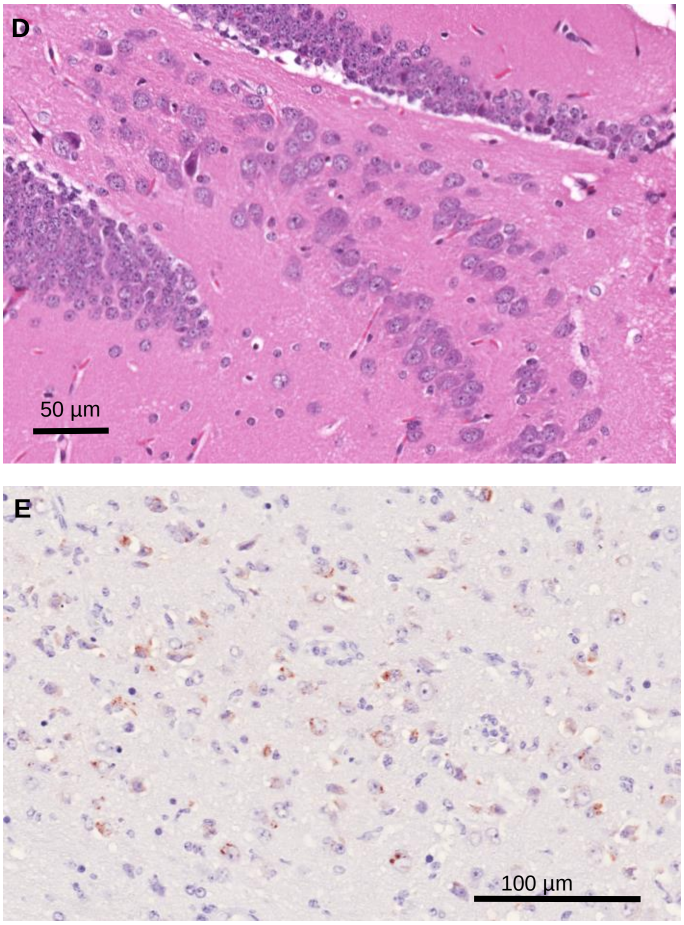

Supplementary Fig. 2. Immuno-histochemistry of *Rag1*$^{-/-}$ mouse brain. (A) *Rag1*$^{-/-}$ mouse brain (replicate 1; passage 5), was stained with the pan-flavivirus anti-NS1 monoclonal antibody 4G4 as described (Nguyen et al. 2024. npj Viruses 2, 15). Brown staining indicates NS1 antigen-positive, ZIKV-infected cells in the hypothalamus, particularly Cornu Ammonis region 3 (CA3). Positive cells appear to be neurons based on morphology and location. (B) Negative control, stained as in A.

(C) Left; enlargement of the CA3 region shown in A. Right; parallel section stained by H&E. Arrows indicate examples of histology consistent with neuron vacuolation (hydropic degeneration); a common feature across the CA3 region shown here. (D) H&E staining of CA3 region in a naïve mouse; neuron vacuolation not apparent. (E) IHC staining (same brain as in A) showing ZIKV-infected cells (with neuronal morphology) in a region of the cortex.

A

Replicate #1 - passage 5 *Rag1*$^{-/-}$ serum → C6/36 cells (passage 1) → C6/36 cells (passage 2) → 6 dpi virus stock ZIKV-V153D
Replicate #2 - passage 5 *Rag1*$^{-/-}$ serum → C6/36 cells (passage 1) → C6/36 cells (passage 2) → 6 dpi virus stock ZIKV-N154D
Replicate #3 - passage 5 *Rag1*$^{-/-}$ serum → C6/36 cells (passage 1) → C6/36 cells (passage 2) → 6 dpi virus stock ZIKV-V153I/T156I

B

Replicate from Fig. 2B	Passage in C6/36	Supernantant harvest	Nucleotide sequence	Amino acid sequence	Used for virus stock (Fig. 2C)
#1	2	6 dpi	GATAATGACACA	DNDT	ZIKV-V153D
#1	2	10 dpi	GATAATGACACA	DNDT	
#2	2	6 dpi	GTTGATGACACA	VDDT	ZIKV-N154D
#2	2	10 dpi	GTTGATGACACA	VDDT	
#3	2	6 dpi	ATTAATGACATA	INDI	ZIKV-V153I/T156I
#3	2	10 dpi	ATTAATGACATA	INDI	

C **ZIKV-V153D**

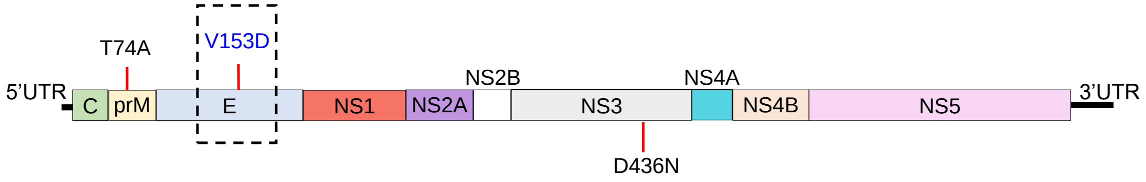

Supplementary Fig. 3. Capillary sequencing details for viral stocks described in Fig. 2C. (A) Passage history to generate C6/36-derived viral stocks after 5 passages in *Rag1*$^{-/-}$ mice. (B) Capillary sequencing results for envelope N-linked glycosylation motif; the 6 dpi data is also shown in Fig. 2C. (C) Mutation map of ZIKV-V153D stock virus after passage in C6/36 cells and capillary sequencing of all mutation sites shown in Fig. 2A, Replicate #1. V153D enriched to ~100%. T74A and D436N enriched to ~100%. Of the remaining partial substitutions (Fig. 2A, Replicate #1): 44% V21C, reverted to ~100% V21; 44% C36Y reverted to ~100% C36; and 23% R545K reverted to ~100% R545. T74A has been identified previously as a naturally occurring substitution in 12 out of 344 sequences but has, to the best of our knowledge, not been ascribed a particular function or activity (1). To the best of our knowledge there are no previous reports describing a D436N substitution in the NS3 protein.

1. Gupta Y, Baranwal M, Chudasama B. 2023. Immunoinformatics-Based Identification of the Conserved Immunogenic Peptides Targeting of Zika Virus Precursor Membrane Protein. Viral Immunol 36:503-519.

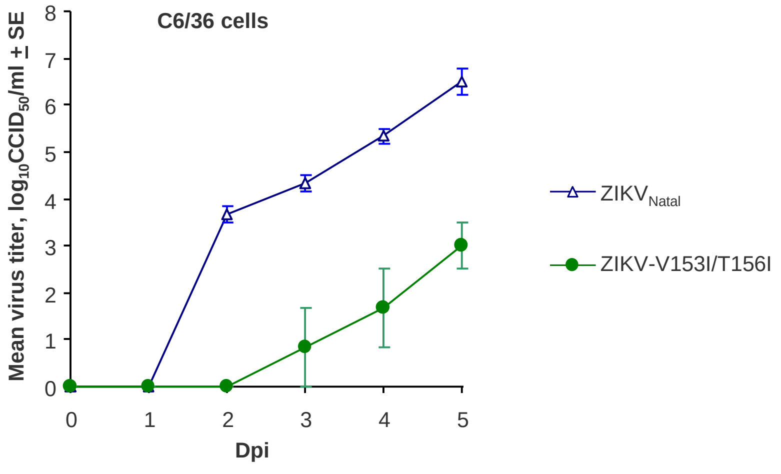

Supplementary Fig. 4. Growth kinetic in C6/36 cells, as described for Fig. 3B. $ZIKV_{Natal}$ is the same data as in Fig. 3B.

A

ZIKV-N154D
4 dpi in organoids

	Position	Natal reference	Change	Allele frequency	Total read depth	
**N17K**	524	C	A	0.97	13789	
**V50A**	1126	T	C	0.38	13792	**Glycosylation motif**
	1437	A	G	0.99	15379	**N154D**
	3392	C	T	0.17	12477	
	4328	C	T	0.96	9562	
	4574	A	G	1	12848	
	4910	C	T	0.19	10033	
**T174P**	5133	A	C	0.99	8909	
	6140	A	G	0.47	12798	
	6242	T	C	0.99	12237	
	6908	T	C	0.99	13584	
	7727	G	A	1	10481	
**G129R**	8052	G	A	0.96	7788	
	9644	A	G	0.99	11683	
	10223	C	T	0.94	8768	

B

$ZIKV_{Natal}$
4 dpi in organoids

	Position	Natal reference	Change	Allele frequency	Total read depth
**G29A**	1062	G	A	0.17	1418
	4328	C	T	0.84	5030
	4574	A	G	1.00	6707
	5900	C	T	0.39	7097

Supplementary Fig. 5. RNA-Seq of human brain organoids 4 dpi analysed for viral SNPs. (A) ZIKV-N154D. The glycosylation motif mutation was retained (see Fig. 4B). Also retained and enriched (from Fig. 2A, Replicate #2, after 2 passages in C6/36 cells, and one passage in the organoids) were N17K, T174P and G129R. A new non-synonymous substitution was identified after the latter passages, V50A at 38%, which was not seen in virus from *Rag1*$^{-/-}$ mouse serum. Other changes (black text) represent synonymous changes. (B) $ZIKV_{Natal}$. One non-synonymous substitution was detected at a frequency of 17%, G29A.

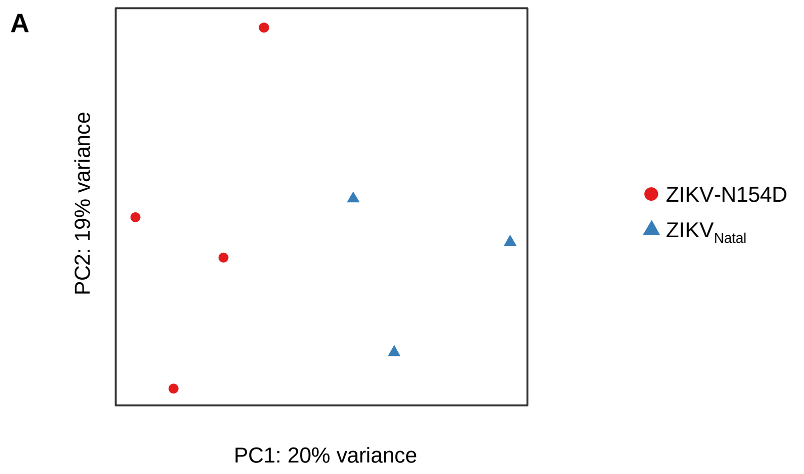

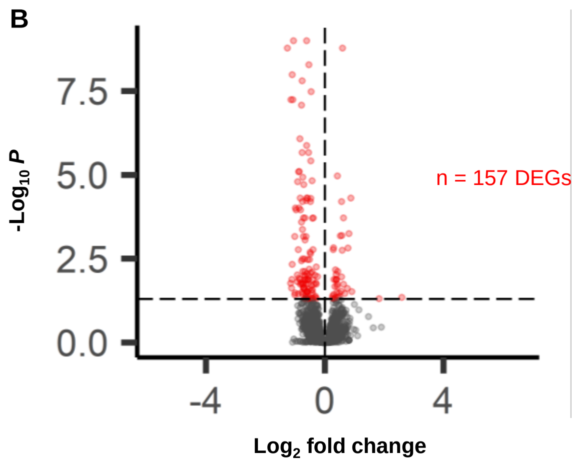

Supplementary Fig. 6. (A) PC2/PC1 plot for human brain organoid RNA-Seq, ZIKV-N154D vs. $\mathrm{ZIKV}_{\mathrm{Natal}}$, 4 dpi. (B) Volcano plot of gene expression pattern for ZIKV-N154D vs. $\mathrm{ZIKV}_{\mathrm{Natal}}$, 4 dpi.

ZIKV$_{\mathrm{Natal}}$ infected *Ifnar1$^{-/-}$* mice, spleen 3 dpi

GAGGCTGGGGAAATGGATGTGGACTTTTTGGCAAAGGG
AGCCTGGTGACATGCGCTAAGTTTGCATGCTCCAAGAAAATGACCGGGAAGAGCATCCAG
CCAGAGAATCTGGAGTACCGGATAATGCTGTCAGTTCATGGCTCCCAGCACAGTGGGATG
ATC**GTTAATGACACA**GGACATGAAACTGATGAGAATAGAGCGAAGGTTGAGATAACGCCC
AATTCACCAAGAGCCGAAGCCACCCTGGGGGGTTTTGGAAGCCTAGGACTTGATTGTGAA
CCGAGGACAGGCCTTGACTTTTCAGATTTGTATTACTTGACTATGAATAACAAGC

n=3, all 3 mice returned the same sequence (RT PCR and capillary sequencing)

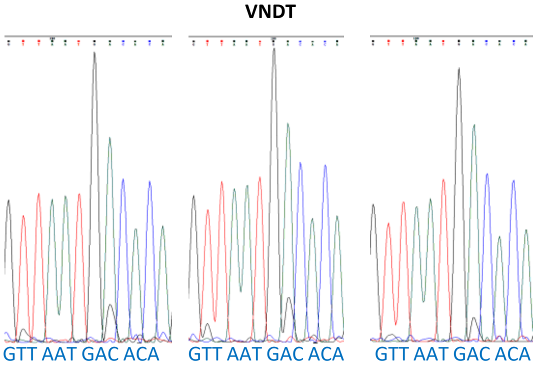

ZIKV-V153D infected *Ifnar$^{-/-}$* mice, spleen 3 dpi

AGTGGACAGAGGCTGGGGAAATGGATGTGGACTTTTTGGCAAAGGGA
GCCTGGTGACATGCGCTAAGTTTGCATGCTCCAAGAAAATGACCGGGAAGAGCATCCAGC
CAGAGAATCTGGAGTACCGGATAATGCTGTCAGTTCATGGCTCCCAGCACAGTGGGATG
ATCGTTAATGACACAGGACATGAAACTGATGAGAATAGAGCGAAGGTTGAGATAACGCCCA
………**A** ……………………………………………………………………………………………………
ATTCACCAAGAGCCGAAGCCACCCTGGGGGGTTTTGGAAGCCTAGGACTTGATTGTGAAC
CGAGGACAGGCCTTGACTTTTCAGATTTGTATTACTTGACTATGAATAACAAGCACTGGT

n=3, all 3 mice returned the same sequence (RT PCR and capillary sequencing)

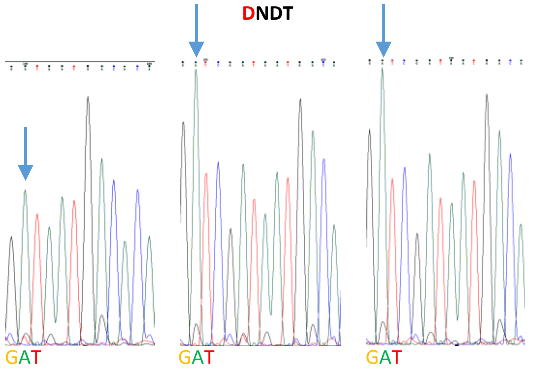

ZIKV-N154D infected *Ifnar1$^{-/-}$* mice, spleen 3 dpi

TTAGTGGACAGAGGCTGGGGAAATGGATGTGGACTTTTTGGCAAAGGG
AGCCTGGTGACATGCGCTAAGTTTGCATGCTCCAAGAAAATGACCGGGAAGAGCATCCAG
CCAGAGAATCTGGAGTACCGGATAATGCTGTCAGTTCATGGCTCCCAGCACAGTGGGATG
ATCGTTAATGACACAGGACATGAAACTGATGAGAATAGAGCGAAGGTTGAGATAACGCCC
. . . .**G**... . . . .
AATTCACCAAGAGCCGAAGCCACCCTGGGGGGTTTTGGAAGCCTAGGACTTGATTGTGAA
CCGAGGACAGGCCTTGACTTTTCAGATTTGTATTACTTGACTATGAATAACAA

n=3, all 3 mice returned the same sequence (RT PCR and capillary sequencing)

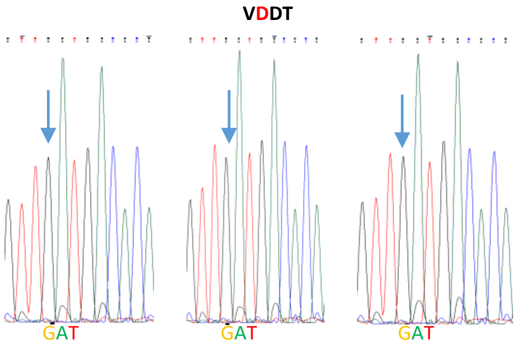

Supplementary Fig. 7. No reversion to wild-type glycosylation motif (top) at 3 dpi of *Ifnar1$^{-/-}$* mice infected with ZIKV-V153D or ZIKV-N154D. Data derived from Nanopore sequencing.

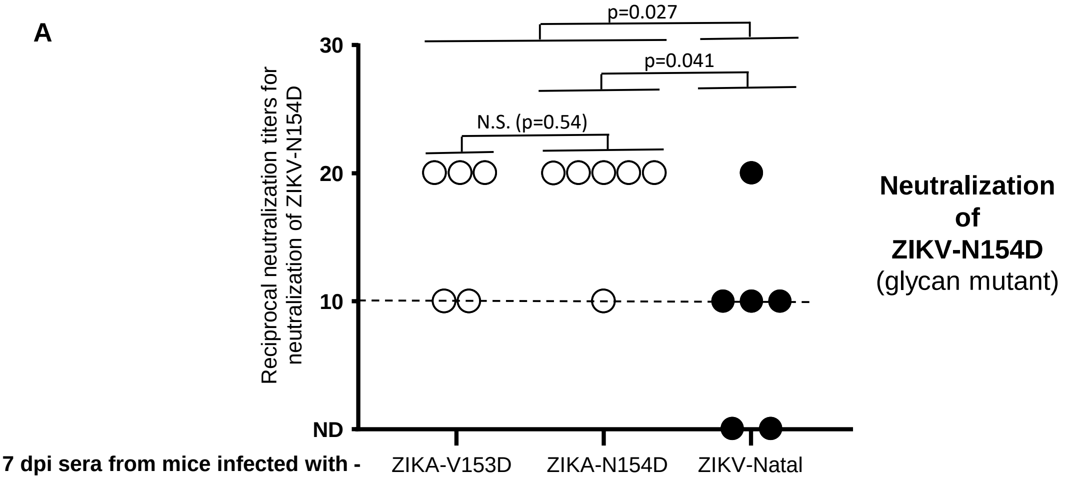

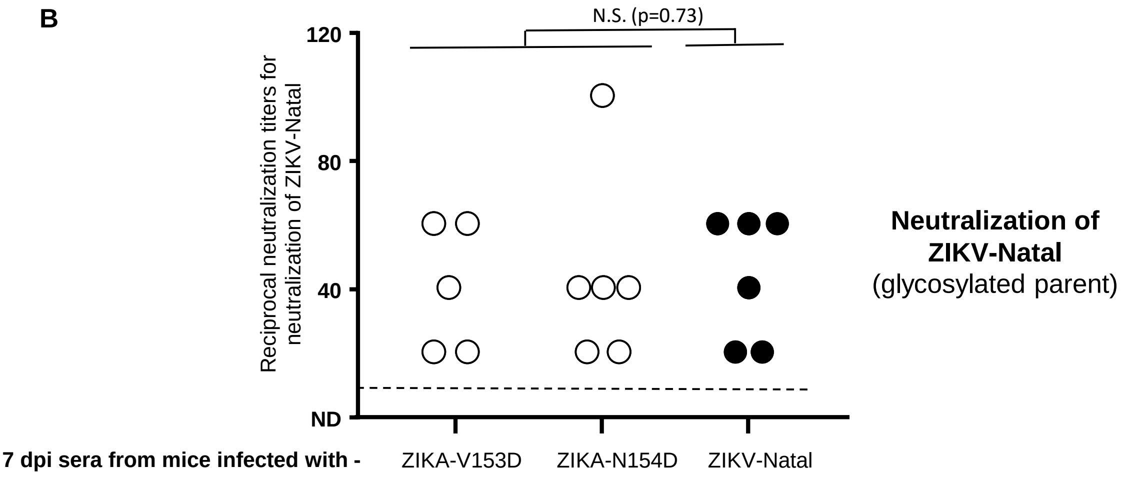

Supplementary Fig. 8. (A) Sera taken from *Ifnar1*$^{-/-}$ mice 7 dpi (Fig. 5A) infected with the glycan mutants, ZIKA-V153D and ZIKA-N154D, were significantly better at neutralizing the glycan mutant, ZIKV-N154D, than was sera taken 7 dpi from mice infected with ZIKV-Natal. This might arise from induction of higher neutralizing antibody levels and/or because the glycan mutants are more readily neutralized by antibodies generated in mice infected with the glycan mutants. Dashed line - limit of detection, serum dilution of 1 in 10 (mean of duplicates); ND – not detected. Statistics by Mann Whitney U tests. (B) In contrast, neutralizing antibody titers for neutralization of the glycosylated virus, ZIKV-Natal, were not significantly different for the different sera. Neutralization titers cannot be compared with those shown in A as ZIKV-Natal replicates much less efficiently in the Vero E6 indicator cells (Fig. 3C) making it "easier" to neutralize in this assay system. Dashed line - limit of detection serum dilution of 1 in 10. Statistics by Mann Whitney U test.

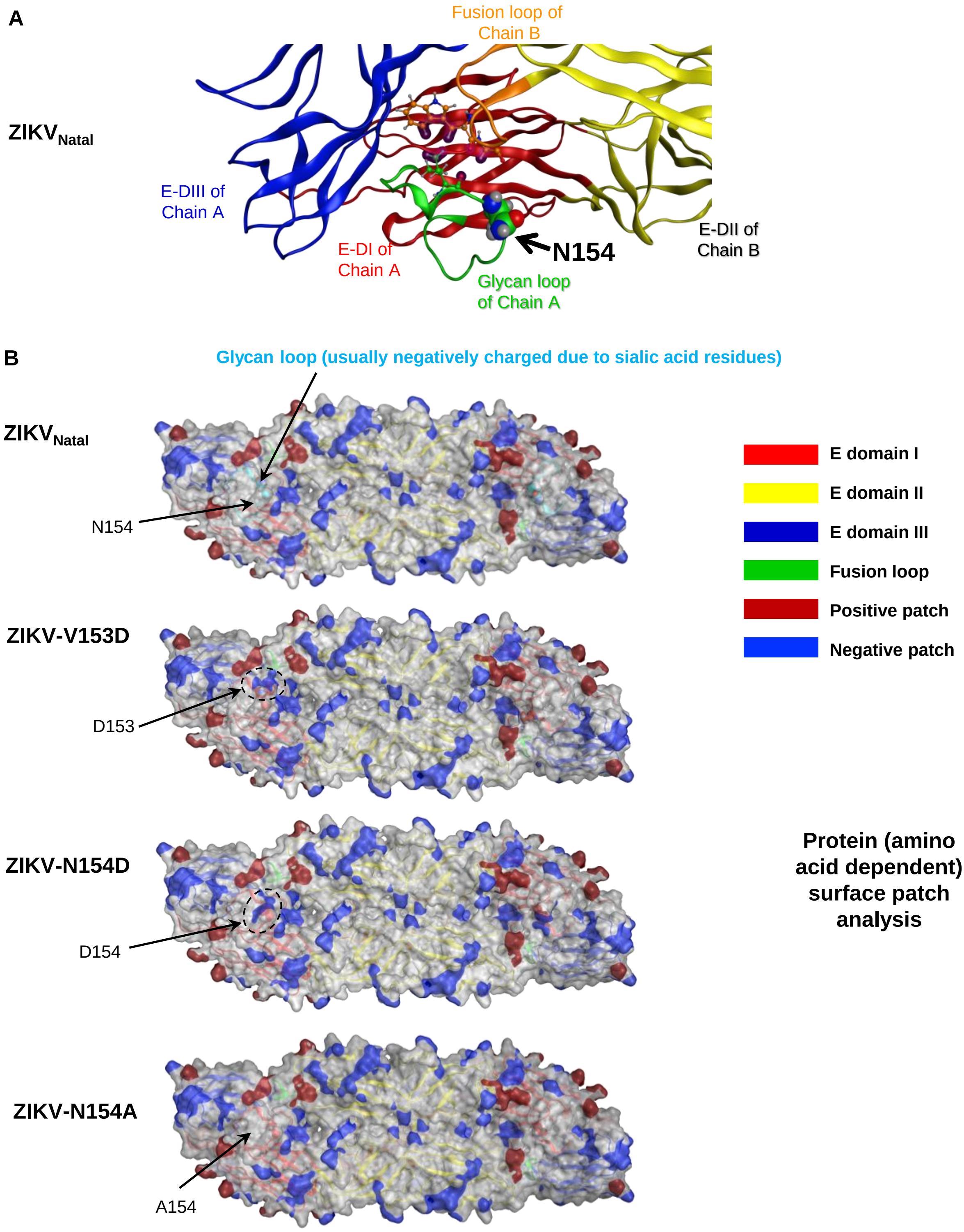

Supplementary Fig. 9. Structure of ZIKV envelope protein Protein Data Base: 5IRE analyzed using the Molecular Operating Environment (MOE) 2022.02 (Chemical Computing Group, Inc., Montreal, Quebec, Canada). (A) Ribbon structure illustrating the close proximity of the glycan loop and the fusion loop in wild-type ZIKV such as $ZIKV_{Natal}$. (B) Protein surface patch analysis conducted in the setting of the Amber10:EHT force field and the generalized Born/volume integral (GB/VI) implicit solvent model. Glycan charge features are not incorporated into the program, with ZIKV glycans usually containing negatively charged sialic acid residues. For ZIKV-V153D and ZIKV-N154D the glycan loop is lost, but the region retains a positive surface charge due to the D mutations (dashed ovals). In contrast, ZIKV-N154A, has no surface positive charge in this region.
