# Supplementary material for: Evolution of Zika virus in Rag1-deficient mice selects for unique envelope glycosylation motif mutants that show enhanced replication fitness
Source: Virus Evol. 2025 Apr 11;11(1):veaf021. doi: 10.1093/ve/veaf021 (PMC12024116; doi:10.1093/ve/veaf021)
Supplement: veaf021_Supp [file veaf021_supp.zip › suppl_data/Supplementary Table 1.pdf]

Supplementary Table 1. Studies of flavivirus envelope N-linked glycosylation.

**References reporting that flavivirus envelope glycan mutants show reduced *in vitro* replication in mammalian cells**		
**Reference**	**Virus mutation**	**Effect of glycan loss**
Ishida *et al*. 2023 (1)	JEV N154A	Lower viral titers in Huh7 Vero, SH-SY5Y, HeLa, C6/36, and 293T cells. Reduced virus secretion into cell culture supernatant via inhibition of ER-Golgi transition.
Liu *et al*. 2021 (2)	DTMUV (duck Tembusu virus) N154Q and N154I	Reduced replication Duck Embyo Fibroblasts (DEFs) and C6/36 cells.<br><br>Reduced infection and mortality in 3 day old ducks after intracerebral infection.
Adam *et al*. 2021 (3)	ZIKV N154Q + T156V	Lower replication kinetics in Vero and A549 cells.<br><br>Reduced neurovirulence in Swiss Webster mice.<br>Reduced viremia and mortality in four week old AB6 mice.<br>Reduced infection of mosquitoes *Ae.aegypti*..
Gwon *et al*. 2020 (4)	ZIKV N154Q	Reduced virus replication in Vero cells. Reduced envelope protein secretion into cell culture supernatant.
Carbaugh *et al*. 2019 (5)	ZIKV N154Q, or T156I	Reduced replication in A549 cells. No difference in virus replication in Vero cells.<br><br>Attenuated upon subcutaneous but not intracranial inoculation as measured by lower viremia, lower brain, serum and eye titers and lower mortality in *Ifnar*$^{-/-}$ mice.
Liang *et al*. 2018 (6)	JEV N154A	Reduced virus growth kinetics on human neuroblastoma HTB-11 cells. Smaller plaques in BHK-21 cells.<br><br>Reduced mortality in 5 week old C57BL/6 mice (both i.c. and i.p.).
Alen *et al*. 2012 (7)	DENV-2 N67D and T155I	Reduced replication in BHK-21, Huh-7 and Vero cells.<br><br>Higher infection of C6/36 cells
Mossenta *et al*. 2017 (8)	ZIKV T156I	Compromised expression and secretion of envelope ectodomain from mammalian cells. Reduced packaging into pseudoviral particles with a West Nile virus replicon system
Liu JV 2015	JEV N154A	Reduced viral titers in BHK-21 cells.
Yoshii *et al*. 2013 (9)	TBEV N154Q	Lower virus replication and smaller plaque size in BHK cells.<br><br>No difference in ISE6 cells (tick cells). Reduced mortality in 5 week old C57BL/6 mice after subcutaneously inoculation. Lower viremia level and virus load in spleen and brain.
Zai *et al*. 2013 (10)	JEV N154Q	Reduced the envelope protein folding efficiency and secretion.
Prow *et al*. 2011 (11)	MVEV 154-deletion	Delay in virus replication in PSEK cells.

		Higher survival rates in 3 weeks old Swiss mice after intraperitoneal infection. Higher infection doses of the mutated strain cause less mortality. Less infectious virus detected from infected mice tissues.
Whiteman *et al*. 2010 (12)	WNV N154S or Gly-/-	Reduced growth kinetics in Neuro2A cells.<br><br>Reduced neuroinvasive and neurovirulence in 5 weeks old NIH Swiss mice after intraperitoneal infection.<br>Reduced viremia on day 2 and day 3 post infection.
Lee *et al*. 2010 (13)	DENV-2 T155A	Lower viral replication in BHK cells and Vero cells.<br><br>Higher infectivity and virus release in C6/36 cells.<br>*Note: no error bars or statistics.
Bryant *et al*. 2007 (14)	DENV N153Q	Reduced replication in Vero cells.<br><br>Lower peak virus titers in C6/36 cells. No growth property difference in mosquitoes.
Mondotte *et al*. 2007 (15)	DENV N153Q	Reduced virus replication in BHK cells.<br><br>No replication difference in C6/36 cells.
Shirato *et al*. 2006 (16)	WNV S156P	Less replication in Vero E6 cells.<br><br>No virus replication difference in murine macrophages.
Li *et al*. 2006 (17)	WN(S)V-S154A	Less virus replication in Vero cells, BHK-21 cells and C6/36 cells.
Beasley *et al*. 2005 (18)	WNV: NY99 Gly-/Gly+	Reduced growth in Vero cells.<br><br>Reduced vireamia and brain infection in 3 to 4 weeks NIH Swiss mice after intraperitoneal infection.
Goto *et al*. (19)	TBEV N154Q	Reduced replication in 293T cells. E protein retained in the endoplasmic reticulum.
Shirato *et al*. 2004 (20)	WNV S156F	Reduced replication in BHK cells.<br><br>Reduced mortality and earlier clinical signs of disease in 6 weeks old BALB/c mice after s.c infection.
Scherret *et al*. 2001 (21)	WNV (KUN) F156S	Reduced growth in Vero and C6/36 cells.<br><br>No clear correlation between neuroinvasiveness and glycosylation status in mice.
Moudy *et al*. 2009(22)	WNV N154I	Reduced replication in Vero and DF-1 cells.<br><br>No difference in C6/36 cells, but reduced replication in *Cx, pipiens* and *Cx. Tarsalis*

References reporting that envelope N-linked glycan mutations had mixed or no effect on replication *in vitro* in mammalian cells		
Yan *et al.* 2018 (23)	TMUV (Tembusu virus) S156P	No change in replication in DF-1 cells (chicken embryo fibroblasts).<br><br>Reduced viremia, lung, kidney and ovary titers in ducks. Ablates transmission (duck-to-duck contact).
Annamalai *et al.* 2017 (24)	ZIKV 153-VNDT-156 deletion, or N154A	No difference in virus replication in Vero or C6/36.<br><br>Attenuated virulence after subcutaneous infection of A129 mice.
Cheng *et al.* 2022 (25)	ZIKV glycan loop deletions 153-VNDT-156 and 153- VNDTGH-158, or T156I	No difference in growth kinetics in BHK-21, Vero or C6/36.<br><br>Deletions reduced oral mosquito infection levels. Deletions had lower viremia and mortality in adult A129 mice injected subcutaneously. Deletions had increased viremia and morality in neonatal Balb/c mice injected intracerebrally.
Maharaj *et al.* 2019 (26)	WNV 154-NYS-156 versus NYT, NYP, NYF, SYS, SYP, KYS, or deletion	Some glycan mutants showed increased replication in HD11 cells (avian monocytes) whereas others showed reduced replication.
Frumence *et al.* 2019 (27)	ZIKV I152T, T156I and H158Y	No difference in virus replication in Vero cells.
Fontes-Garfias *et al.* 2017 (28)	ZIKV N154Q	No difference in Vero or BHK cells. Better infection of primary mouse dendritic cells.<br><br>Improved attachment and assembly in C6/36 cells.<br>Lower viremia and mortality in 3 week old A129 mice (subcutaneous infection). No difference in mortality after intracranial infection of 1 day old CD-1 mice.<br>Lower oral infection rate of *A. aegypti*.
Hanna J. Virol 2005	WNV N154Q	Modest effect on replication in mammalian (BHK-21) or avian (QT6) cells, reduced release of WNV subviral particles.<br><br>Increase infectivity on mosquito cells.
Alsaleh *et al.* 2016 (29)	WNV E/Gly(-) or S156P	No *in vitro* data.<br><br>Limited impact on viral virulence

References

1. Ishida K, Yagi H, Kato Y, Morita E. 2023. N-linked glycosylation of flavivirus E protein contributes to viral particle formation. PLOS Pathogens 19:e1011681. https://doi.org/10.1371/journal.ppat.1011681.

2. Liu D, Xiao X, Zhou P, Zheng H, Li Y, Jin H, Jongkaewwattana A, Luo R. 2021. Glycosylation on envelope glycoprotein of duck Tembusu virus affects virus replication in vitro and contributes to the neurovirulence and pathogenicity in vivo. Virulence 12:2400-2414. https://doi.org/10.1080/21505594.2021.1974329.
3. Adam A, Fontes-Garfias CR, Sarathy VV, Liu Y, Luo H, Davis E, Li W, Muruato AE, Wang B, Ahatov R, Mahmoud Y, Shan C, Osman SR, Widen SG, Barrett ADT, Shi P-Y, Wang T. 2021. A genetically stable Zika virus vaccine candidate protects mice against virus infection and vertical transmission. npj Vaccines 6:27. https://doi.org/10.1038/s41541-021-00288-6.
4. Gwon Y-D, Zusinaite E, Merits A, Överby AK, Evander M. 2020. N-glycosylation in the Pre-Membrane Protein Is Essential for the Zika Virus Life Cycle. Viruses 12:925. https://doi.org/10.3390/v12090925.
5. Carbaugh DL, Baric RS, Lazear HM. 2019. Envelope Protein Glycosylation Mediates Zika Virus Pathogenesis. Journal of Virology 93:e00113-19. https://doi.org/10.1128/jvi.00113-19.
6. Liang J-J, Chou M-W, Lin Y-L. 2018. DC-SIGN Binding Contributed by an Extra N-Linked Glycosylation on Japanese Encephalitis Virus Envelope Protein Reduces the Ability of Viral Brain Invasion. Frontiers in Cellular and Infection Microbiology 8:239. https://doi.org/10.3389/fcimb.2018.00239.
7. Alen MM, Dallmeier K, Balzarini J, Neyts J, Schols D. 2012. Crucial role of the N-glycans on the viral E-envelope glycoprotein in DC-SIGN-mediated dengue virus infection. Antiviral Res 96:280-7. https://doi.org/10.1016/j.antiviral.2012.10.007.
8. Mossenta M, Marchese S, Poggianella M, Slon Campos JL, Burrone OR. 2017. Role of N-glycosylation on Zika virus E protein secretion, viral assembly and infectivity. Biochemical and Biophysical Research Communications 492:579-586. https://doi.org/10.1016/j.bbrc.2017.01.022.
9. Yoshii K, Yanagihara N, Ishizuka M, Sakai M, Kariwa H. 2013. N-linked glycan in tick-borne encephalitis virus envelope protein affects viral secretion in mammalian cells, but not in tick cells. J Gen Virol 94:2249-2258. https://doi.org/10.1099/vir.0.055269-0.
10. Zai J, Mei L, Wang C, Cao S, Fu ZF, Chen H, Song Y. 2013. N-glycosylation of the premembrane protein of Japanese encephalitis virus is critical for folding of the envelope protein and assembly of virus-like particles. Acta Virol 57:27-33. https://doi.org/10.4149/av_2013_01_27.
11. Prow NA, May FJ, Westlake DJ, Hurrelbrink RJ, Biron RM, Leung JY, McMinn PC, Clark DC, Mackenzie JS, Lobigs M, Khromykh AA, Hall RA. 2011. Determinants of attenuation in the envelope protein of the flavivirus Alfuy. J Gen Virol 92:2286-2296. https://doi.org/10.1099/vir.0.034793-0.
12. Whiteman MC, Li L, Wicker JA, Kinney RM, Huang C, Beasley DW, Chung KM, Diamond MS, Solomon T, Barrett AD. 2010. Development and characterization of non-glycosylated E and NS1 mutant viruses as a potential candidate vaccine for West Nile virus. Vaccine 28:1075-83. https://doi.org/10.1016/j.vaccine.2009.10.112.
13. Lee E, Leang SK, Davidson A, Lobigs M. 2010. Both E protein glycans adversely affect dengue virus infectivity but are beneficial for virion release. J Virol 84:5171-80. https://doi.org/10.1128/JVI.01900-09.
14. Bryant JE, Calvert AE, Mesesan K, Crabtree MB, Volpe KE, Silengo S, Kinney RM, Huang CY, Miller BR, Roehrig JT. 2007. Glycosylation of the dengue 2 virus E protein at N67 is critical for virus growth in vitro but not for growth in intrathoracically inoculated Aedes aegypti mosquitoes. Virology 366:415-23. https://doi.org/10.1016/j.virol.2007.05.007.
15. Mondotte JA, Lozach PY, Amara A, Gamarnik AV. 2007. Essential role of dengue virus envelope protein N glycosylation at asparagine-67 during viral propagation. J Virol 81:7136-48. https://doi.org/10.1128/JVI.00116-07.

16. Shirato K, Miyoshi H, Kariwa H, Takashima I. 2006. The kinetics of proinflammatory cytokines in murine peritoneal macrophages infected with envelope protein-glycosylated or non-glycosylated West Nile virus. Virus Res 121:11-6. https://doi.org/10.1016/j.virusres.2006.03.010.
17. Li J, Bhuvanakantham R, Howe J, Ng ML. 2006. The glycosylation site in the envelope protein of West Nile virus (Sarafend) plays an important role in replication and maturation processes. J Gen Virol 87:613-622. https://doi.org/10.1099/vir.0.81320-0.
18. Beasley DW, Whiteman MC, Zhang S, Huang CY, Schneider BS, Smith DR, Gromowski GD, Higgs S, Kinney RM, Barrett AD. 2005. Envelope protein glycosylation status influences mouse neuroinvasion phenotype of genetic lineage 1 West Nile virus strains. J Virol 79:8339-47. https://doi.org/10.1128/JVI.79.13.8339-8347.2005.
19. Goto A, Yoshii K, Obara M, Ueki T, Mizutani T, Kariwa H, Takashima I. 2005. Role of the N-linked glycans of the prM and E envelope proteins in tick-borne encephalitis virus particle secretion. Vaccine 23:3043-3052. https://doi.org/https://doi.org/10.1016/j.vaccine.2004.11.068.
20. Shirato K, Miyoshi H, Goto A, Ako Y, Ueki T, Kariwa H, Takashima I. 2004. Viral envelope protein glycosylation is a molecular determinant of the neuroinvasiveness of the New York strain of West Nile virus. J Gen Virol 85:3637-3645. https://doi.org/10.1099/vir.0.80247-0.
21. Scherret JH, Mackenzie JS, Khromykh AA, Hall RA. 2001. Biological significance of glycosylation of the envelope protein of Kunjin virus. Ann N Y Acad Sci 951:361-3. https://doi.org/10.1111/j.1749-6632.2001.tb02719.x.
22. Moudy RM, Zhang B, Shi PY, Kramer LD. 2009. West Nile virus envelope protein glycosylation is required for efficient viral transmission by Culex vectors. Virology 387:222-8. https://doi.org/10.1016/j.virol.2009.01.038.
23. Yan D, Shi Y, Wang H, Li G, Li X, Wang B, Su X, Wang J, Teng Q, Yang J, Chen H, Liu Q, Ma W, Li Z. 2018. A Single Mutation at Position 156 in the Envelope Protein of Tembusu Virus Is Responsible for Virus Tissue Tropism and Transmissibility in Ducks. Journal of Virology 92:10.1128/jvi.00427-18. https://doi.org/10.1128/jvi.00427-18.
24. Annamalai AS, Pattnaik A, Sahoo B, R., Muthukrishnan E, Natarajan SK, Steffen D, Vu H, Delhon G, Osorio FA, Petro TM, Xiang S-H, Pattnaik AK. 2017. Zika Virus Encoding Nonglycosylated Envelope Protein Is Attenuated and Defective in Neuroinvasion. Journal of Virology 91:e01348-17. https://doi.org/10.1128/jvi.01348-17.
25. Cheng M-L, Yang Y-X, Liu Z-Y, Wen D, Yang P, Huang X-Y, Dong H-L, Xu Y-P, Li X-F, Deng Y-Q, Ye Q, Zhu L, Li J, Davidson Andrew D, Zheng A-H, Shi W-F, Zhao H, Wang X-X, Qin C-F. 2022. Pathogenicity and Structural Basis of Zika Variants with Glycan Loop Deletions in the Envelope Protein. Journal of Virology 96:e00879-22. https://doi.org/10.1128/jvi.00879-22.
26. Maharaj PD, Langevin SA, Bolling BG, Andrade CC, Engle XA, Ramey WN, Bosco-Lauth A, Bowen RA, Sanders TA, Huang CYH, Reisen WK, Brault AC. 2019. N-linked glycosylation of the West Nile virus envelope protein is not a requisite for avian virulence or vector competence. PLOS Neglected Tropical Diseases 13:e0007473. https://doi.org/10.1371/journal.pntd.0007473.
27. Frumence E, Viranaicken W, Bos S, Alvarez-Martinez M-T, Roche M, Arnaud J-D, Gadea G, Desprès P. 2019. A Chimeric Zika Virus between Viral Strains MR766 and BeH819015 Highlights a Role for E-glycan Loop in Antibody-mediated Virus Neutralization. Vaccines 7:55. https://doi.org/https://doi.org/10.3390/vaccines7020055.
28. Fontes-Garfias CR, Shan C, Luo H, Muruato AE, Medeiros DBA, Mays E, Xie X, Zou J, Roundy CM, Wakamiya M, Rossi SL, Wang T, Weaver SC, Shi P-Y. 2017. Functional Analysis of Glycosylation of Zika Virus Envelope Protein. Cell Reports 21:1180-1190. https://doi.org/https://doi.org/10.1016/j.celrep.2017.10.016.

29. Alsaleh K, Khou C, Frenkiel MP, Lecollinet S, Vazquez A, de Arellano ER, Despres P, Pardigon N. 2016. The E glycoprotein plays an essential role in the high pathogenicity of European-Mediterranean IS98 strain of West Nile virus. Virology 492:53-65. https://doi.org/10.1016/j.virol.2016.02.009.
