# Supplementary material for: Evolution of Zika virus in Rag1-deficient mice selects for unique envelope glycosylation motif mutants that show enhanced replication fitness
Source: Virus Evol. 2025 Apr 11;11(1):veaf021. doi: 10.1093/ve/veaf021 (PMC12024116; doi:10.1093/ve/veaf021)
Supplement: veaf021_Supp [file veaf021_supp.zip › suppl_data/Supplementary Table 2.pdf]

Name	Sequence
ZIKVNatal_1_Fwd	TTTCTGTTGGTGCTGATATTGCTGAATCAGACTGCGACAGTTCG
ZIKVNatal_1_Rev	ACTTGCCTGTCGCTCTATCTTCCCAAGTCCCACCTGACATACCT
ZIKVNatal_2_Fwd	TTTCTGTTGGTGCTGATATTGCTTGGCTTTTGGGAAGCTCAACG
ZIKVNatal_2_Rev	ACTTGCCTGTCGCTCTATCTTCCCTTCCCTTTGCACCATCCATC
ZIKVNatal_3_Fwd	TTTCTGTTGGTGCTGATATTGCTTCAAGGACGCACATGCCAAAA
ZIKVNatal_3_Rev	ACTTGCCTGTCGCTCTATCTTCATACCATCTTCCCAGGCTTGCT
ZIKVNatal_4_Fwd	TTTCTGTTGGTGCTGATATTGCCGGTACAGGGGTGTTCGTCTAT
ZIKVNatal_4_Rev	ACTTGCCTGTCGCTCTATCTTCCAGTTGATCCTGCAGTCACCAC
ZIKVNatal_5_Fwd	TTTCTGTTGGTGCTGATATTGCCTGTCGTTCCGGGCTAAAGATG
ZIKVNatal_5_Rev	ACTTGCCTGTCGCTCTATCTTCACACTCTTTCCTGAGACCACGT
ZIKVNatal_6_Fwd	TTTCTGTTGGTGCTGATATTGCCTGTTGGCCTGATATGCGCATT
ZIKVNatal_6_Rev	ACTTGCCTGTCGCTCTATCTTCCTTCTTCAGCATCGAAGGCTCG
ZIKVNatal_7_Fwd	TTTCTGTTGGTGCTGATATTGCGGACTTTATGGCAATGGGGTCG
ZIKVNatal_7_Rev	ACTTGCCTGTCGCTCTATCTTCTGCATGGTCTTCGTCAGTCTCT
ZIKVNatal_8_Fwd	TTTCTGTTGGTGCTGATATTGCGAGAGTCATTTTGGCTGGACCC
ZIKVNatal_8_Rev	ACTTGCCTGTCGCTCTATCTTCGCGGTAATCAAGCCCAGAAGAC
ZIKVNatal_9_Fwd	TTTCTGTTGGTGCTGATATTGCGTTCCTATTGCTGGTGGTGCTC
ZIKVNatal_9_Rev	ACTTGCCTGTCGCTCTATCTTCCCTTCCATTTCTCTCCCAGGGT
ZIKVNatal_10_Fwd	TTTCTGTTGGTGCTGATATTGCAGGGGAAGTTACTTGGCTGGAG
ZIKVNatal_10_Rev	ACTTGCCTGTCGCTCTATCTTCTCACTGCGGATCCTTTCAATGC
ZIKVNatal_11_Fwd	TTTCTGTTGGTGCTGATATTGCTGAGGAGGATGTGAATCTCGGC
ZIKVNatal_11_Rev	ACTTGCCTGTCGCTCTATCTTCGCCCTTTCTCCATTTGGTTGGT
ZIKVNatal_12_Fwd	TTTCTGTTGGTGCTGATATTGCTGAGTCGCATACCAGGAGGAAG
ZIKVNatal_12_Rev	ACTTGCCTGTCGCTCTATCTTCTCCACACCACAAGTCTTCCCTT
ZIKVNatal_13_Fwd	TTTCTGTTGGTGCTGATATTGCACAAGCTCCATCTCAAGGACGG
ZIKVNatal_13_Rev	ACTTGCCTGTCGCTCTATCTTCCGTGGTGGAAACTCATGGAGTC

Supplementary Table 2. ZIKV primers used for Nanopore sequencing.
