# Supplementary material for: Evolution of Zika virus in Rag1-deficient mice selects for unique envelope glycosylation motif mutants that show enhanced replication fitness
Source: Virus Evol. 2025 Apr 11;11(1):veaf021. doi: 10.1093/ve/veaf021 (PMC12024116; doi:10.1093/ve/veaf021)
Supplement: veaf021_Supp [file veaf021_supp.zip › suppl_data/Supplementary Table 3.pdf]

		MR766	ZIKV Natal				Replicate #1				Replicate #2				Replicate #3			
		amino acid	A	C	G	T	A	C	G	T	A	C	G	T	A	C	G	T
Nucleotide #	169					92%		44%		49%				92%				
Amino acid #	C - 21	V				V		C		V				V				
Nucleotide #	475			94%												24%		71%
Amino acid #	prM - 1	A		A												A		S
Nucleotide #	524			98%				98%			93%							
Amino acid #	prM - 17	S		N				N			K							
Nucleotide #	693		89%		10%		11%		88%		87%		12%					
Amino acid #	prM - 74	T	T		A		T		A		T		A					
Nucleotide #	1241					96%			46%	48%				96%				
Amino acid #	E - 88	T				T			T	T				T				
Nucleotide #	1263					98%		35%		64%				98%				
Amino acid #	E - 96	L				L		L		L				L				
Nucleotide #	1434				96%										89%		9%	
Amino acid #	E - 153	V			V										I		V	
Nucleotide #	1434					97%	40%			57%				95%				
Amino acid #	E - 153	V				V	D			V				V				
Nucleotide #	1437		97%				48%		52%		6%		94%					
Amino acid #	E - 154	N	N				N		D		N		D					
Nucleotide #	1444			92%		7%										10%		89%
Amino acid #	E - 156	T		T		I										T		I
Nucleotide #	2066			69%		30%		67%		31%		69%		30%				
Amino acid #	E - 363	P		P		P		P		P		P		P				
Nucleotide #	3083		94%		4%										12%		87%	
Amino acid #	NS1 - 198	L	L		V										L		V	
Nucleotide #	3218					98%									69%			31%
Amino acid #	NS1 - 243	I				I									I			I
Nucleotide #	3392			98%				98%				74%		26%				
Amino acid #	NS1 - 301	T		T				T				T		T				
Nucleotide #	3791		98%												11%		87%	
Amino acid #	NS2A - 82	A	A												A		A	
Nucleotide #	3970					99%										41%		57%
Amino acid #	NS2A - 142	M				M										T		M
Nucleotide #	3989					97%		25%		74%				98%				
Amino acid #	NS2A - 148	D				D		D		D				D				
Nucleotide #	4083					96%										89%		8%
Amino acid #	NS2A - 180	I				F										L		F
Nucleotide #	4152			96%												4%		95%
Amino acid #	NS2A - 203	L		L												L		L
Nucleotide #	4328			15%		84%		3%		95%		3%		95%				
Amino acid #	NS2B - 1407	V		V		V		V		V		V		V				
Nucleotide #	4574		6%		93%		6%		93%		6%		93%					
Amino acid #	NS2B - 1489	A	A		A		A		A		A		A					
Nucleotide #	4795		91%		6%		58%		38%		90%		6%					
Amino acid #	NS3 - 1587	L	L		L		L		L		L		L					
Nucleotide #	4910			95%				95%				71%		27%				
Amino acid #	NS3 - 1601	A		A				A				A		A				
Nucleotide #	5036			96%				72%		25%		96%						
Amino acid #	NS3 - 1643	D		D				D		D		D						
Nucleotide #	5133		95%				95%				7%	89%						
Amino acid #	NS3 - 1676	T	T				T				T	P						
Nucleotide #	5477			98%				39%		60%		98%						
Amino acid #	NS3 - 1790	H		H				H		H		H						
Nucleotide #	5657			98%												30%		67%
Amino acid #	NS3 - 1856	G		G												G		G
Nucleotide #	5900			49%		50%										15%		84%
Amino acid #	NS3 - 1931	C		C		C										C		C
Nucleotide #	5919				97%		35%		63%				98%					
Amino acid #	NS3 - 1938	D			D		N		D				D					
Nucleotide #	6242					97%				97%		89%		9%				
Amino acid #	NS3 - 2045	V				V				V		V		V				
Nucleotide #	6429			97%												16%		82%
Amino acid #	NS3 - 2108	L		L												L		L
Nucleotide #	6566		97%				77%		22%		97%							
Amino acid #	NS4A - 2153	A	A				A		A		A							
Nucleotide #	6987		93%		6%										13%		84%	
Amino acid #	NS4B - 2294	T	T		A										T		A	
Nucleotide #	7727		7%		91%		8%		91%		93%		6%					
Amino acid #	NS5 - 2540	S	L		S		L		S		L		S					
Nucleotide #	7774				98%		44%		55%				98%					
Amino acid #	NS5 - 2556	C			C		Y		C				C					
Nucleotide #	7860			4%		95%										68%		30%
Amino acid #	NS5 - 2585	L		L		L										L		L
Nucleotide #	7870		36%		59%		36%		59%		36%		60%		34%		61%	
Amino acid #	NS5 - 2588	R	Q		R		Q		R		Q		R		Q		R	
Nucleotide #	8052		11%		87%		11%		87%		81%		17%					
Amino acid #	NS5 - 2649	G	R		G		R		G		R		G					
Nucleotide #	8270				99%										90%		9%	
Amino acid #	NS5 - 2721	R			R										R		R	
Nucleotide #	8402			97%												5%		92%
Amino acid #	NS5 - 2765	D		D												D		D
Nucleotide #	8873			98%												37%		62%
Amino acid #	NS5 - 2922	N		N												N		N
Nucleotide #	9301				97%		23%		76%				97%					
Amino acid #	NS5 - 3065	K			R		K		R				R					
Nucleotide #	9644		96%				96%				5%		95%					
Amino acid #	NS5 - 3179	R	R				R				R		R					
Nucleotide #	10026				97%										67%		29%	
Amino acid #	NS5 - 3307	V			V										I		V	
Nucleotide #	10121				98%		38%		61%				98%					
Amino acid #	NS5 - 3338	V			V		V		V				V					

KEY TO TABLE
White = No change
Yellow = Difference between MR766 and Natal
Red border = N-linked glycosylation site

	% of mutations	Total mutations
Green = non-synonymous	45.2	19
Blue = synonymous	54.8	23
**Total**	**100.0**	**42**

Supplementary Table 3. Nucleotide and amino acid changes in the viruses in the serum of *Rag1*$^{-/-}$ mice at the end of passage 5. There are three Replicate series, with changes indicated relative to the input parental $ZIKV_{Natal}$.
